# Gut Microbiota Dysbiosis: Pathogenesis, Diseases, Prevention, and Therapy

**DOI:** 10.1002/mco2.70168

**Published:** 2025-04-18

**Authors:** Yao Shen, Nairui Fan, Shu‐xia Ma, Xin Cheng, Xuesong Yang, Guang Wang

**Affiliations:** ^1^ International Joint Laboratory for Embryonic Development & Prenatal Medicine Division of Histology and Embryology School of Medicine Jinan University Guangzhou China; ^2^ Key Laboratory for Regenerative Medicine of the Ministry of Education Jinan University Guangzhou China; ^3^ Basic Medical College of Jiamusi University Heilongjiang China; ^4^ International School Guangzhou Huali College, Zengcheng Guangzhou China; ^5^ Guangdong‐Hong Kong Metabolism & Reproduction Joint Laboratory Guangdong Second Provincial General Hospital School of Medicine Jinan University Guangzhou China

**Keywords:** gut microbiota, dysbiosis, disease, precision medicine

## Abstract

Dysbiosis refers to the disruption of the gut microbiota balance and is the pathological basis of various diseases. The main pathogenic mechanisms include impaired intestinal mucosal barrier function, inflammation activation, immune dysregulation, and metabolic abnormalities. These mechanisms involve dysfunctions in the gut–brain axis, gut–liver axis, and others to cause broader effects. Although the association between diseases caused by dysbiosis has been extensively studied, many questions remain regarding the specific pathogenic mechanisms and treatment strategies. This review begins by examining the causes of gut microbiota dysbiosis and summarizes the potential mechanisms of representative diseases caused by microbiota imbalance. It integrates clinical evidence to explore preventive and therapeutic strategies targeting gut microbiota dysregulation, emphasizing the importance of understanding gut microbiota dysbiosis. Finally, we summarized the development of artificial intelligence (AI) in the gut microbiota research and suggested that it will play a critical role in future studies on gut dysbiosis. The research combining multiomics technologies and AI will further uncover the complex mechanisms of gut microbiota dysbiosis. It will drive the development of personalized treatment strategies.

## 1 Introduction

The human gut serves an anaerobic bioreactor, hosting a wide variety of microorganisms, including bacteria, fungi, archaea, protozoa, and viruses and others, which are collectively termed the microbiota. They occupy different ecological niches on the mucosal surface of the gastrointestinal (GI) tract [1]. The microorganisms in the gut make up most of the human microbiome, including at least 1000 different species of bacteria and approximately 150 times the number of genes in the human genome [2]. The coevolution between the human host and microorganisms has established a mutually beneficial symbiotic relationship. The immense genetic and metabolic potential of the gut microbiota makes it nearly ubiquitous. The host provides a suitable environment and nutrients for the microbiota, while the microbiota plays a significant role in the host's homeostasis and disease.

The characteristics of a healthy gut microbiome are a diversified and balanced microbial community that performs important functions for the host. The gut dysbiosis is usually defined as a decrease in microbial diversity, an absence of beneficial microbiotas, or an increase in harmful microbiotas. The biological significance of the gut microbiome is evident from the early stages of life. Postnatal development of the gut microbiota contributes to shape the neonatal immune system [3, 4]. Later, it plays a critical role in various physiological processes, such as maintaining homeostasis, participating in immune regulation, and modulating the central nervous system (CNS) and enteric nervous system (ENS) [5, 6]. With the advancement of microbiome bioinformatics, research on the microbiome has deepened. Many studies have reported that changes in the gut microbiota occur not only during obesity, diabetes, and liver disease, but also during cancer and even neurodegenerative diseases. The bidirectional communication between microorganisms, the gut, and multiple systems such as microbiome–gut–brain axis [7], microbiome–gut–liver axis [8], enhances clinical comprehension of disease progression. The microbiome's dynamic and diverse characteristics, along with its responsiveness to external inputs, underscores its potential as a novel target for therapeutic interventions [9, 10].

Given the critical role of the gut microbiome in health and disease, this review aims to summarize the pathogenic mechanisms of gut microbiota dysbiosis and updated relationship between gut microbiota dysbiosis and the development of diseases. It will provide insights into the treatment of gut microbiota dysbiosis and related diseases. The highlight of this review lies in its integration of multifaceted research on gut microbiota dysbiosis, providing an in‐depth exploration of the mechanisms underlying different diseases and existing intervention strategies. while also envisioning the potential impact of combining multiomics technologies and artificial intelligence (AI) on future gut microbiota research. We hope that this review will provide insights for further research in this field and offer scientific evidence for clinical applications, driving the personalized development of prevention and treatment strategies for gut microbiota‐related diseases.

This review will begin by exploring the pathogenic mechanisms that trigger gut microbiota dysbiosis, discuss the relationship between gut microbiota dysbiosis and various diseases (such as enteritis, obesity, diabetes, neurological disorders, etc.), and then summarize current prevention and treatment methods, such as probiotics, prebiotics, and fecal microbiota transplantation (FMT). Especially, we will focus on the development of emerging therapeutic strategies, including the application of traditional Chinese medicine (TCM), as well as how precise interventions can improve the research progress in gut microbiota dysbiosis. Finally, discuss the challenges and future directions in gut microbiota research, with a particular focus on the potential applications of multiomics technologies and AI in this field.

## 2 Pathogenesis of Gut Microbiota Dysbiosis

The human gut microbiome is a complex ecosystem, densely colonized by thousands of microbial species. It varies between individuals and is constantly influenced by host genetic and environmental factors, which affect the composition and functional profile. The diversity, metabolic flexibility, functional coordination, and interactions among microbes–microbes and microbes–host in the gut microbiota are crucial for maintaining healthy homeostasis. Due to the combination of natural variations and stress factors can lead to a series of unstable changes, the potential mechanisms of gut microbiota dysbiosis remain unclear. This section will explore the main factors that lead to gut microbiota dysbiosis and analyze the potential mechanisms by which dysbiosis induces disease, with a focus on the roles of microbial metabolite imbalance, impaired intestinal barrier function, and immune system dysregulation.

### 2.1 Factors Contributing to Dysbiosis

#### 2.1.1 Diet and Lifestyle

The initial colonization of gut microbiota in early life is determined by the mother, but subsequent changes are more influenced by environmental factors. Diet and lifestyle are recognized as critical determinants of gut microbiota composition [11, 12]. Changes in host nutrition induce temporary shifts in microbial composition, but major components such as meat, fish, and fiber can have a lasting effects, leaving characteristic specific bacterial patterns [13]. For example, a high‐calorie, high‐fat, low‐fiber diet increases the abundance of *Proteus species*. Excessive intake of food additives can also impair blood glucose control and induce the proliferation of Proteus [14]. A long‐term Mediterranean diet can partially mitigate age‐related changes in gut microbiota composition and metabolic function, particularly unhealthy aging [15, 16]. Growing evidence shows that the composition and function of the gut microbiota are altered in obese individuals in both humans and rodents. FMT can shape host metabolism and impact obese phenotypes [17, 18, 19], demonstrating that diet‐induced gut microbiota dysbiosis is a major cause of the disease rather than a consequence. In clinical trial cases, a low‐fat and high‐fiber diet strategy in ulcerative colitis (UC) patients can reduce inflammatory markers in the feces of patients and alleviate gut dysbiosis [20]. After a low‐fat, high‐fiber diet, the abundance of Bacteroides significantly increased, while Actinobacteria decreased in the feces of UC patients. The increase in *Faecalibacterium prausnitzii* and other microbes led to an anti‐inflammatory shift in the microbiome. These findings support that long‐term appropriate dietary interventions will be an effective approach to regulating gut microbiota dysbiosis.

#### 2.1.2 Antibiotics and Other Medications

Antibiotic drugs have an inherent potential to promote dysbiosis through their antimicrobial activity. The most common effect of antibiotics on the gut microbiome are decreased phylogenetic diversity and richness. The increase abundance of *Proteobacteria*, including *Enterobacteriaceae*, leading to a proinflammatory state and enhanced bacteria expression of antibiotic resistance genes. Excessive antibiotic exposure in early life may have lasting negative effects on gut microbiota immunity, metabolism, and endocrinology. These effects could persist for years, potentially lasting into adulthood [21]. The use of macrolide antibiotics in children leads to a long‐term reduction in *Firmicutes* and *Actinobacteria*, while increasing *Bacteroidetes* and *Proteobacteria*. Additionally, the recovery time of the microbiome's homeostasis is associated with the frequency of antibiotic use. Long time use may result in lasting alterations in the microbiota composition [22]. Antibiotic‐induced dysbiosis can even alter the host transcriptome and m6A epitranscriptomic modifications through its metabolites [23]. Furthermore, exposure to other drugs or exogenous compounds can also cause gut microbiota dysbiosis [24]. In most cases, we only observe the correlation between exposure and disease outcomes, while the specific changes in the microbiota and their mechanisms of action still require further investigation. Although antibiotics are an important and essential medical tool, society must currently emphasize the long‐term impacts of antibiotic overuse. Avoiding unnecessary antibiotic use is crucial for maintaining the ecological balance of the gut microbiota and preserving overall health.

#### 2.1.3 Infections and Inflammation

Inflammation is the body's normal protective defense response to infection or injury. It is a driving factor for intestinal permeability and microbial dysbiosis. Pathogen infections, such as viruses and bacteria, can cause intestinal inflammation lead to dysbiosis. During the body's homeostasis, the gut microbiota maintains a diverse population of beneficial microorganisms (symbionts) to produce a balanced immune response. However, proinflammatory microbiota (pathogenic bacteria) may decrease or increase during dysbiosis. This changes shifting the balance between proinflammatory and anti‐inflammatory responses toward an inflammatory phenotype associated with various diseases, such as multiple sclerosis (MS) and inflammatory bowel disease (IBD) [25]. Generally, Intestinal immunity stimulates the occurrence of intestinal inflammation and protects the body from harmful pathogens. But excessive activation of Th cells promotes the progression of intestinal inflammation [26]. Infection by foreign microorganisms trigger changes in the composition of the microbiota may cause a series of events. The enrichment of pathogenic bacteria and the release of harmful toxins lead to proinflammatory environment and impaired intestinal barrier function [27]. For example, *Klebsiella pneumoniae* crosses the mucosal layer to invade intestinal epithelial cells. And then activate host macrophages to release inflammatory cytokines such as interleukin (IL)‐1β and tumor necrosis factor‐α (TNF‐α). This heightened proinflammatory state disrupts intestinal homeostasis, leading to an imbalance in the microbial environment [28].

#### 2.1.4 Host Genetic Predisposition

In addition to environmental factors, host‐specific factors (such as host genetics) can influence the composition of the gut microbiota. Some genetic variations in the host may make individuals more susceptible to dysbiosis, which is an important factor in the development of metabolic and immune‐related diseases. A study analyzing the gut microbiome and human variation in the TwinsUK cohort, which included 250 individuals, identified microbiome‐related metabolic traits (e.g., body mass index and blood pressure) as well as common microbial functions (such as secretion system proteins and antibiotic resistance) associated microbiome‐related diseases. For example type 2 diabetes mellitus (T2DM) and some neurological disorders were associated with host genetics. The genetic loci of solute carrier family 22 member 5 (*SLC22A5)*, G protein‐coupled receptor 35 (*GPR35)*, and *GPR65* are associated with the risk of IBD onset and host–microbe interactions [29]. Genetic similarities in the microbiome have been identified in twin and familial population genetic studies, particularly in the phyla *Firmicutes* and Verrucomicrobia [30]. Besides, variants in certain individual genes can directly affect the composition of the gut microbiota. For example, the lactase (LCT)locus is associated with *Actinobacteria* and *Bifidobacterium*, while interactions exist between ABO and fucosyltransferase 2 (FUT2) variants and bacterial abundance [31]. Establishing the relationship between host genetic susceptibility and gut microbiota dysbiosis is instrumental in understanding the relationship between gut microbiota imbalance and the development of diseases. On the one hand, genetic variations may directly lead to disease phenotypes, which in turn can trigger changes in the microbiome. On the other hand, they may directly alter the microbiome lead to disease phenotypes. Microbiome‐wide association genetic studies are expected to identify additional host genetic variations that influence disease progression by disrupting the microbiome composition. It will provide a clearer explanation of the interplay between genetic susceptibility and gut microbiota dysbiosis.

#### 2.1.5 Differences in Intestinal Flora Among Different Populations

An early study based on metagenomic sequencing analysis compared the gut microbiome composition of populations from different geographical regions (such as the United States, Europe, Africa, and Asia) [32]. Although all populations share a common microbiome, there are significant differences in the gut microbiome details across different regions. These differences may be closely related to factors such as dietary habits, environmental factors, and genetic background. In addition, differences in gut microbiome composition are also observed across different age groups, with gut microbiome diversity significantly decreasing as age increases [33]. Infants have a characteristic microbiome before weaning, which then transforms into a more diverse microbiome with the introduction of solid foods. As individuals age, the microbiome becomes relatively stable until late adulthood (around 65 years), after which diversity peaks and begins to decline, becoming more pronounced in individuals over 80 years old [34]. In the elderly population, changes in a diverse microbiome (including organisms suspected of producing anti‐inflammatory metabolites such as SCFAs) are closely associated with enhanced inflammatory responses and the occurrence of chronic diseases. Others, the composition of the gut microbiome also shows significant differences in populations with unhealthy conditions. For example, obese patients have lower gut microbiome diversity and a specific microbial composition, such as an imbalance in the ratio of *Firmicute*s to *Bacteroidete*s [35], which also highlights the potential link between gut microbiome dysbiosis and obesity. Besides, compared with healthy controls, specific types of bacteria (such as certain anaerobes) are more abundant in diabetic patients [36], and the dysbiosis of these microbial populations is closely associated with insulin resistance and metabolic abnormalities.

Existing research on gut microbiome dysbiosis mostly focuses on specific populations or single disease models, lacking cross‐sectional comparative studies on dysbiosis across different populations or conditions. Incorporating cross‐population studies can significantly enhance the understanding of how gut microbiome dysbiosis varies across different genetic backgrounds, lifestyles, dietary habits, and environmental conditions. This will help reveal the general patterns of gut microbiome dysbiosis and its impact on the health of different populations.

### 2.2 Mechanisms of Dysbiosis‐Induced Disease

**FIGURE 1:**
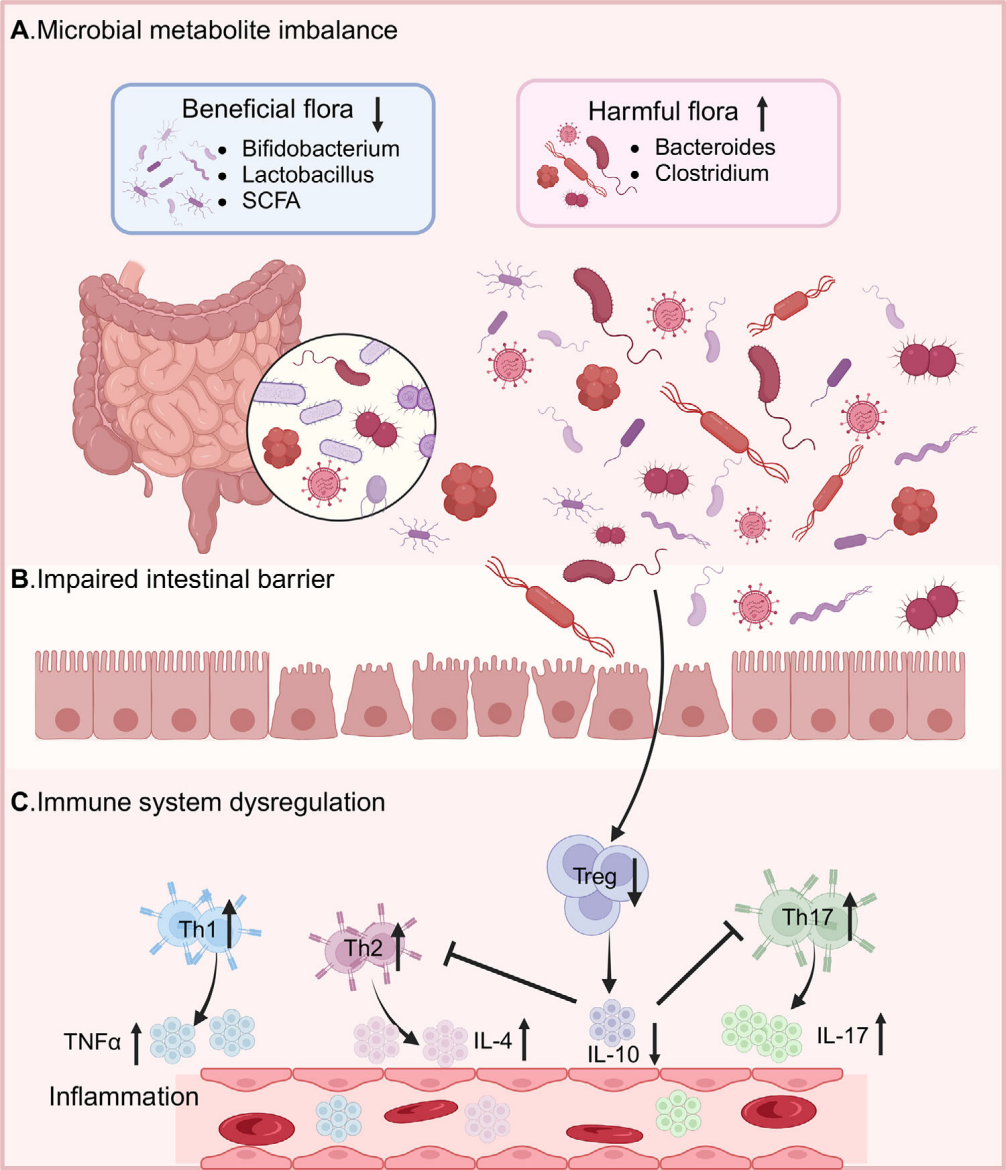
The mechanism by which gut microbiota dysbiosis triggers diseases. (A) Dysbiosis of the gut microbiota leads to an imbalance in microbial metabolism and metabolites. The reduction of beneficial bacteria and an increase in opportunistic pathogens disrupt the microbial functions necessary for maintaining health. (B) The expansion of opportunistic pathogens leading to the disruption of intestinal barrier. Intestinal pathogens through translocation and dissemination released into the circulation, resulting in systemic immune dysfunction. C: The increase in opportunistic pathogens activates T helper cells (Th1, Th2, and Th17 cells), leading to the release of inflammatory factors. The reduction of beneficial bacteria inhibits the formation of Treg cells, further exacerbating the inflammatory response. *Abbreviations*: TNFα, tumor necrosis factor α; IL, interleukin; Treg, T regulatory cells (created in BioRender. Yang, X. (2025) https://BioRender.com/undefined).

The homeostasis of the gut microbiota plays a crucial role in maintaining normal human health and has a wide range of effects [37]. When the body experiences gut microbiota dysbiosis, it may increase the likelihood of various diseases such as GI disorders, neurological diseases, and metabolic conditions. It has been found that gut microbiota dysbiosis may increase the likelihood of disease development through mechanisms such as microbial metabolic imbalance, impaired gut barrier function, and immune dysregulation (Figure 1).

#### 2.2.1 Microbial Metabolite Imbalance

Dysbiosis of the gut microbiota not only leads to changes in the composition of the microbial community but also results in alterations of metabolites. The gut microbiota can regulate the body's nervous system by influencing the gut–brain axis. The study found that a humid and hot environment induces gut microbiota dysbiosis, such as a decrease in the abundance of *Lactobacillus murinus*, which causes an increase in the levels of secondary bile acids (BAs). This in turn triggers the elevated expression of proinflammatory cytokines and increased neuroinflammation, ultimately promoting the progression of anxiety disorders [38]. The genus *Akkermansia* is a key factor in the production of SCFAs. Gut microbiota dysbiosis causing a reduction in *Akkermansia*, resulting in decreased SCFA synthesis. The reduced abundance of *Akkermansia* and SCFAs increases the likelihood of adults developing hypertension and cardiovascular diseases [39]. Besides, the dysregulation of the gut microbiota and the subsequent reduction in SCFA synthesis promote the onset of neurological diseases. The decrease in butyrate among the SCFAs increases inflammation in the brain and inhibits microglial maturation. Those guide the oligomerization of amyloid‐beta (Aβ) 1–40, which contributes to the development of Alzheimer's disease (AD) [40]. The deficiency of propionate inhibits the survival of dopaminergic cells and the growth of neurites. Furthermore, increasing neuroinflammation promote the onset and progression of Parkinson's disease (PD) [41]. Tryptophan is an important metabolite derived from gut microbiota, aberrantly activates the AHR signaling pathway as a ligand. It will influence the progression of cardiovascular diseases and chronic kidney disease [42]. Indolepropionic acid (IPA) is a tryptophan metabolite involved in renal immunoregulation in hypertensive patients. When IPA is reduced in the dysbiosis, it leads to an increase in Th17 cells and decrease in T‐regulatory (Treg) cells, ultimately leading to elevated blood pressure [43]. In addition, dysbiosis of the gut microbiota inhibits the production of vitamin D3, thereby leading to hypertension [44].

#### 2.2.2 Impaired Intestinal Barrier Function

A healthy human gut has a certain degree of permeability. This property allow nutrients to pass through while maintaining its barrier function to prevent potentially harmful substances from leaving the gut and spreading throughout the body. When the gut microbiota is dysregulated, it can lead to increased intestinal permeability, triggering the development of GI diseases. Intestinal barrier dysfunction is referred to as “leaky gut” or intestinal permeability syndrome. Commonly, disruption of intestinal barrier integrity is one of the characteristics of IBD. The study found that a decrease in SCFAs caused by gut microbiota dysbiosis leads to increased intestinal permeability. This change increases the likelihood of developing IBD [45]. Moreover, the increased intestinal permeability is closely associated with the later stages of Crohn's disease (CD) progression [46].The impairment of gut barrier function not only affects GI disorders but also impacts CNS diseases [47]. Increased intestinal immune/inflammatory responses caused by intestinal flora imbalance can damage the integrity and permeability of the intestinal barrier function. Next, it triggers CNS inflammation and neurodegenerative processes contribute to the occurrence of PD [48]. A high‐fat diet can impair intestinal barrier integrity in mice, leading to changes in glial cells, a reduction in dendritic spines of neurons, and behavioral changes consistent with depression and anxiety phenotypes [49]. Intestinal barrier disruption can affect the function of microglial cells in the CNS through the gut–brain axis, leading to the onset of MS [50]. In addition, due to the disruption of the intestinal barrier, inflammatory responses occur in the mesenteric lymph nodes, which cause the liver inflammation. Persistent hepatic inflammation can trigger excessive activation and proliferation of hepatic stellate cells to form liver fibrosis [51]. Clinical studies have shown that the loss of barrier integrity is closely associated with metabolic disorders, including obesity and T2DM [52]. At the same time, it will lead to metabolic endotoxemia, which is considered a major factor in insulin resistance and obesity [53].

#### 2.2.3 Immune System Dysregulation

The disruption of the intestinal barrier caused by gut microbiota dysbiosis further triggers an imbalance in the intestinal microenvironment's immune response. This change contributes to the development of various diseases with the uncontrolled growth of harmful microorganisms. Changes in the gut microbiota are associated with various systemic autoimmune diseases. Dysbiosis of the gut microbiota causes an increase in parasitic bacteria (*segmented filamentous bacteria*), which trigger arthritis by promoting the elevation of Th17 cells and the production of autoantibodies [54]. Additionally, *Cellulomonas flava* (CFB) affects the Th17/Treg balance to influence immune homeostasis and contributes to autoimmune diseases [55]. Type 1 diabetes (T1DM) is a systemic autoimmune disease associated with microbial dysbiosis. It is characterized by an abundance of bacteria that produce acetate and propionate in the gut microbiota, which in turn impairs neutrophil function in patients with T1DM [56]. Besides, gut microbiota dysbiosis plays a potential role in the development of MS. The increase of proinflammatory bacteria induces Th1/Th17 differentiation, which can systematically spread to the brain and lead to an increase in inflammatory factors [57]. The symptoms of autoimmune diseases can be partially improved by giving probiotics and prebiotics to mice. Therefore, understanding the impact of gut microbiota on immune system dysregulation plays an important role in the treatment of diseases (Table 1).

**TABLE 1:** Mechanisms of dysbiosis‐induced disease.

Disease type	Mechanisms related to dysbiosis	References
**Gastrointestinal diseases**		
IBD	Gut microbiota dysbiosis and abnormal bile acid metabolism	[58]
IBS	Microbial metabolites impair insulin sensitivity	[59]
UC	Sphingolipid metabolism disorders	[60]
CRC	Abnormal bile acid metabolism, especially taurodeoxycholic acid (DCA), and intestinal barrier disruption	[61]
CRC	High doses of soluble fiber induce intestinal flora imbalance, decrease in probiotic *Bifidobacterium pseudolongum* and its metabolite inosine, accompanied by increases in fecal butyrate and serum bile acid	[62]
Colitis	*Enterococcus* mediated intestinal barrier damage	[63]
Colitis	Intestinal barrier destruction and immune homeostasis disorder	[64]
Cholestatic liver injury	*Lactobacillus acidophilus* ameliorates cholestatic liver injury through inhibiting bile acid synthesis and promoting bile acid excretion	[65]
UC	The abundance of *Ruminococcaceae* is reduced, and the expression of abnormal bile acid metabolism genes is reduced.	[66]
Alcoholic hepatitis	Intestinal inflammation caused by dysbiosis activates tumor necrosis factor‐1	[67]
**Neurological disorders**		
AD	Bile acid metabolism disorders	[68]
AD	*Bacteroides fragilis* activates microglia and stimulates immune responses	[69]
AD	Antibiotic ABX‐mediated gut microbiota dysbiosis promotes microglial activation and aggravates Aβ amyloidosis	[70]
AD	Enrichment of *Dubosiella* impedes AD progression via palmitoleic acid synthesis	[71]
		
PD	Intestinal dysbiosis disrupts healthy flora and Th17 homeostatic immunity in the ileal mucosa, leading to a cascade effect that spreads to the brain	[72]
PD	Gut microbiota affects neuroinflammation through SCFA metabolism	[73]
*C90RF72* ALS/FTD	Neuroinflammation	[74]
Depression	Tryptophan metabolism disorders	[75]
Depression	Proline metabolism disorders	[76]
Depression, anxiety	Reduced bacterial flora and reduced tryptophan synthesis	[77]
**Metabolic diseases**		
T2DM	Bile acid metabolism and insulin sensitivity	[78]
T2DM	Gut microbiota abundance and bile acid metabolism	[79]
T2DM combined with MASLD	Impaired intestinal barrier integrity and host–microbiome interactions	[80]
NAFLD	Microbiome‐derived ethanol promotes NFLD	[81]
NAFLD, stem cell cancer	Gut microbiota regulates peripheral immune responses	[82]
NAFLD, liver fibrosis	The metabolic function of the intestinal flora changes, *Bacteroidetes* is independently associated with NASH, while *Ruminococcus* is associated with severe fibrosis	[83]
Obesity	Intestinal microbes degrade inositol and promote lipid absorption	[84]
Obesity	Enterotype‐like microbiota *Megamonas* degrades inositol and promotes lipid absorption	[85]
Obesity	*Akkermansia* disappears and declines, intestinal barrier damage, and metabolic inflammation	[19]
Obesity	Absence of Tlr9 in B cells causes disturbance of intestinal flora	[86]
**Other**		
CSU	Low‐diversity gut microbiota reduces short‐chain fatty acid production and increases lipopolysaccharide levels, promoting mast cell‐driven skin inflammation	[87]
Allergic disease	Dysbiosis promotes immune response	[88]
Atopic dermatitis	Arachidonic acid‐induced intestinal flora imbalance in infants	[89]
Atherosclerosis	*Andida albicans* activates intestinal hypoxia‐inducible factor 2α	[90]
Mastitis	Modulates inflammatory processes and regulates blood‐milk barrier permeability	[91]
Mastitis	Gut microbiota dysbiosis leads to endotoxemia, thereby reducing host anti‐inflammatory enzyme activity	[92]
Hypertension	Overgrowth of bacteria such as *Prevotella* and *Klebsiella*	[93]

*ABbreviations*: IBD: inflammatory bowel disease; IBS: irritable bowel syndrome; UC: ulcerative colitis; AD: Alzheimer's disease; PD: Parkinson's disease; ALS: amyotrophic lateral sclerosis; FTD: frontotemporal dementia; T2DM: type 2 diabetes mellitus; MASLD: metabolic dysfunction‐related fatty liver; NAFLD: nonalcoholic fatty liver disease; CSU: chronic spontaneous urticaria.

## 3 Diseases Associated with Gut Microbiota Dysbiosis

### 3.1 GI Diseases

The microbial community in the human gut is extremely diverse with approximately 100 trillion microorganisms [35]. Many studies have revealed the important relationship between the gut microbiota and fundamental biological processes in humans. Under healthy conditions, the gut microbiota exhibits stability, resilience, and a symbiotic relationship with the host. In the coevolutionary process of vertebrates and microorganisms, stable and complex internetwork systems have formed. When this homeostatic balance is disrupted, the GI response is the first to be affected. Microbial imbalance leads to the overgrowth of harmful bacteria, which produce toxic metabolites that damage the intestinal barrier, allowing harmful substances to leak into the bloodstream and trigger local or systemic inflammatory responses. Meanwhile, gut microbiota dysbiosis may also activate or suppress the immune system, resulting in chronic inflammation and immune dysfunction, thereby promoting the onset of diseases such as irritable bowel syndrome (IBS), IBD, and colorectal cancer (CRC).

#### 3.1.1 Irritable Bowel Syndrome

IBS is a condition that affects intestinal motility, intestinal nerve sensitivity, or the brain–gut interaction in controlling these functions [94]. Although it does not increase mortality, but it significantly reduces the quality of life. Moreover, no clear and effective treatment is available. The study found that IBS patients exhibit lower bacterial diversity, indicating dysbiosis of the gut microbiota. A randomized double‐blind clinical trial evaluated the efficacy of FMT in treating IBS patients. The report indicated that improve gut dysbiosis could alleviate IBS symptoms and improve patients’ quality of life [95]. There is also growing evidence suggesting a causal relationship between the gut microbiome and human metabolic health. Studies on human subjects and preclinical monkey models of metabolic syndrome have indicated that gut bacteria *Ruminococcus gnavus*‐derived tryptamine and phenylethylamine play a pathogenic role in insulin resistance induced by gut microbiota dysbiosis in T2DM and IBS. Tryptamine and phenylethylamine produced by *Ruminococcus gnavus*‐mediated dietary amino acid catabolism impair insulin sensitivity by activating the TAAR1–MAPK/ERK signaling axis. It leads to insulin resistance in gut dysbiosis‐related IBS and T2DM [59]. More and more precise research on different subtypes of IBS has increasingly revealed distinct gut microbiome characteristics. Different subtypes have distinct bacterial characteristics, and the associated functional changes are related to the pathogenesis of IBS. For example, the production pathway of hydrogen sulfide is upregulated in IBS with diarrhea, whereas the biosynthesis pathway of palmitic acid is enhanced in IBS with constipation [96]. These results further support the regulatory role of the gut microbiota in IBS.

#### 3.1.2 Inflammatory Bowel Disease

IBD is a group of heterogeneous chronic inflammatory diseases caused by the interaction of genetic factors, environmental factors, and the gut microbiota. IBD typically including UC and CD [97, 98]. Under healthy physiological conditions, symbiotic microorganisms produce beneficial metabolic products that help maintain an impermeable barrier composed of intact mucosa and epithelium. When genetic/immune driving factors, environmental triggers, and lifestyle/dietary changes lead to the onset of a predisease stage, some symbiotic microorganisms transform into pathogenic bacteria that are better adapted to the ecological niche of the ecosystem. As the disease progresses, IBD patients exhibit active inflammatory mucosal damage. The massive emergence and proliferate of pathogenic bacteria leads to the quantity of symbiotic bacteria significantly decrease and a sharp reduction in beneficial metabolites with persistent inflammatory infiltration. Persistent inflammation and long‐term dysbiosis cause immune imbalance and impaired mucosal healing, thereby sustaining inflammation and dysbiosis in a chronic cycle [99]. The intestinal barrier is mainly composed of digestive fluids, symbiotic bacteria, antimicrobial peptides, epithelial cells, and local immune cells. In the case of IBD, bacteria such as *R. gnavus* will degrade mucins in large amounts and disrupt the integrity of the epithelium. The amplification of *Enterococcus faecalis* promotes the production of matrix metalloproteinases (e.g., gelatinase), which degrade E‐cadherin and impair mucosal integrity. At this point, harmful GI bacteria or related toxins, antigens, and so on break through the barrier and translocate. After being phagocytosed by immune cells, a large amount of lipopolysaccharides (LPS) has being released to guide chronic inflammation [100]. At the same time, gut microbiota dysbiosis leads to an imbalance in microbial metabolites, such as BA, SCFAs, tryptophan, medium‐chain fatty acids, and trimethylamine N‐oxide (TMAO). These metabolites regulate the activation of the NLRP3 inflammasome, the secretion of inflammation‐related cytokines, the differentiation of naïve T cells into Treg or Th17 cells, and the trafficking of lymphocytes to extraluminal sites, thus further mediating the composition of the gut microbiome [101].

#### 3.1.3 Colorectal Cancer

Among GI diseases, CRC as the third most common malignancy globally is closely associated with the gut microbiota. Dysbiosis, specific pathogenic microorganisms, metabolites, virulence factors, and microbial carcinogenic mechanisms all contribute to the occurrence and development of CRC [102, 103]. For example, the pathogenic bacterium *Alistipes* increases, while the probiotic *Parabacteroides distasonis* decreases under a high‐fat diet, leading to impaired gut barrier function. Those changes promote colorectal tumorigenesis in germ‐free mice, indicating that the gut microbiota plays a crucial role in high‐fat diet‐associated CRC development [104]. Besides, gut microbiota metabolites such as taurodeoxycholic acid can activate the carcinogenic MAPK/ERK pathway in colonic epithelial cells to promote colon cell proliferation [61]. SCFAs can antagonize CRC cell proliferation, accelerate its apoptosis, and inhibit inflammation‐related CRC carcinogenic pathways [104]. At the same time, they activate drug‐metabolizing enzymes and inhibit the degradation of primary BAs into secondary BAs, what reduce the burden of carcinogens such as BAs [105]. The functions of SCFAs are various, they can act as tumor suppressors by regulating the expression of oncogenes through epigenetic effects during the progression of CRC [106]. Multiomics studies have also revealed the potential application of gut microbiota metabolites in early CRC screening and diagnosis, including various metabolite combinations such as l‐valine, myristic acid, and others [107, 108, 109]. In recent years, several studies have focused on gut microbiota‐related biomarkers for noninvasive diagnosis of CRC and its precancerous lesions. For example, the abundance of *Fusobacterium nucleatum* is significantly increased in patients with CRC and precursor lesions [110, 111]. Additionally, the DNA of *Fusobacterium nucleatum* in oral saliva is an independent factor for predicting the prognosis of CRC [112]. Gut microbiome biomarkers have potential translational application value in the screening and early diagnosis of CRC.

### 3.2 Metabolic Disorders

Unhealthy lifestyles have always been the main risk factors for metabolic diseases. The metabolic and immune potential of the gut microbiota determines its importance for host health and disease. Gut microbiota influences the host's energy balance, insulin sensitivity, and fat accumulation through its metabolic products. At the same time, gut microbiota dysbiosis may also exacerbate the symptoms or promote the progression of metabolic diseases by altering immune responses and promoting chronic low‐grade inflammation. In recent years, a large of evidence has shown that the gut microbiota and its metabolites play a crucial role in the occurrence and development of various metabolic diseases, such as obesity, T2DM, and noncoholic fatty liver disease (NAFLD).

#### 3.2.1 Obesity and Type 2 Diabetes Mellitus

Rapidly changing lifestyles in recent years have exacerbated obesity and the occurrence of obesity‐related diseases. The changes in the gut microbiome caused by obesity have become a potential driving factor for related comorbidities [113]. Diabetes mellitus (DM) is a group of diseases that affect blood glucose regulation. T1DM is caused by an autoimmune response targeting pancreatic β cells, whereas T2DM is characterized by the body's inability to properly produce or use insulin. In T2DM, the gut microbiota is closely associated with the development of the disease. The study confirmed that the gut microbiota composition of T2DM patients has changed [114], and the use of hypoglycemic medications improved the diversity and richness of the gut microbiota. It played a positive role in the treatment of T2DM patients by enriching the intestinal ecosystem with beneficial bacteria. Therefore, the development of T2DM has a subtle interplay with gut microbiota homeostasis. Compared with healthy person, the abundance of *Firmicutes* and *Clostridium butyricum* was significantly lower in T2DM patients. Additionally, the ratio of *Bacteroidetes* to *Firmicutes* and *Bacteroides‐Prevotella* to *Clostridium coccoides‐Eubacterium* was positively correlated with blood glucose levels [115]. The occurrence and development of T2DM is associated with elevated levels of inflammatory factors, including increased LPS in peripheral blood [116]. LPS can bind to the Toll‐like receptor 4 (TLR4) receptor to trigger macrophage aggregation and activate the NF‐κB signaling pathway. This interaction leads to the release of inflammatory factors and inhibit insulin secretion. In addition, the metabolites SCFAs participate in glucose metabolism pathways through various signals. SCFAs can bind to the free fatty acid receptors FFAR2 or FFAR3 on intestinal L cells, stimulating the release of glucagon‐like peptide 1 (GLP‐1) and peptide YY. It can promote insulin secretion and reduces glucagon levels [117]. When T2DM occurs, low‐grade inflammatory infiltration leads to impaired intestinal barrier function. Butyrate produced by gut microbiota plays a crucial role in maintaining the integrity of the intestinal barrier. SCFAs are also important anti‐inflammatory mediators that can limit autoimmune responses by promoting the generation of regulatory T cells [118]. Therefore, the balance of its metabolic products is easily altered when the dynamic balance of gut microbiota is skewed. This unbalanced state can easily promote the occurrence and progression of T2DM.

#### 3.2.2 NAFLD and Gut–liver Axis

Dysbiosis of the gut microbiota repeatedly occurs in obesity and T2DM, both of which are closely associated with NAFLD. The global burden of NAFLD is primarily caused by the dual epidemics of obesity and T2DM [119]. The comorbidity rate of obesity/T2DM and NAFLD is as high as 70–80%, and the incidence of NAFLD is 100% in obese patients with T2DM [120]. The metabolites of the gut microbiota promote the development of NAFLD through multiple pathways. BAs are involved in NAFLD by regulating hepatic and extrahepatic lipid, carbohydrate, and inflammatory pathways by targeting BA receptors [121]. Evidence suggests that inhibiting the production of ceramide in the intestine can reduce lipid accumulation in the liver, thereby preventing high‐fat diet‐induced NAFLD [122]. Specific bacterial traits directly indicate the persistent enrichment of Proteobacteria in steatosis and nonalcoholic steatohepatitis, directly affecting histidine and its metabolite levels [123]. *Clostridia* and *Lactobacillus* genera also exhibit overlap in NAFLD and T2DM [124]. Overall, dysbiosis directly leads to increased intestinal permeability to bacterial products in metabolic diseases, resulting in higher levels of these products in the systemic circulation. Dysbiosis combined with poor diet can also alter the intraluminal metabolism of substrates such as food. This will increase production of certain SCFAs and consumption of choline to exacerbate the disease progression. Currently, an increasing number of studies focus on the relationship between gut microbiota or its specific metabolites and diseases. For example, histidine is negatively correlated with the level of fatty degeneration, and it is a metabolite with high disease predictive capability [123]. Predicting the relationship between differential microbiota, metabolites, and diseases will contribute to the development of prevention and treatment strategies for such diseases.

The establishment of the gut–liver axis facilitates bidirectional crosstalk between the GI tract and liver metabolism in health and disease. The hepatic portal vein collects blood from the small intestine, large intestine, spleen, and pancreas. It is a hallmark anatomical structure for digestive tract–liver communication. In turn, the liver secretes BAs into the intestine via the biliary system, which are reabsorbed in the intestine. This enable bidirectional communication along the liver–gut axis [125]. For example, the metabolism of BAs by gut microbiota enables their involvement in diverse host regulatory processes and activates innate immune genes in the small intestine, resulting in direct or indirect modulation of the gut microbiota [126]. Gut microbiota dysbiosis and intestinal barrier dysfunction may lead to systemic microbial translocation and entry into the hepatic portal circulation [127] to promote the liver diseases. BAs metabolism in the gut–liver axis is mainly regulated by Farnesoid X receptor (FXR) and G protein‐coupled BA receptor. These receptors have been clinically applied in the treatment of liver‐related diseases. For example, the most widely used FXR agonists primarily include BAs derivatives, steroidal compounds, and nonsteroidal compounds. It activates FXR to inhibit the production and influx of BAs and promotes the outflow, thereby alleviating the excessive accumulation of BAs in the liver [128]. A comprehensive understanding of the interactions within the gut–liver axis plays a positive role in disease treatment to help establish a multidimensional approach to therapy. Scientists no longer limit their focus to a single organ or component but instead adopt a holistic perspective to construct an entire biological network, including the establishment of the gut–liver–brain axis. In the future, under the framework of the macro‐network, more detailed exploration and mechanistic explanations will be needed, supported by high‐quality preclinical studies and clinical randomized controlled trials to further develop targeted therapeutic drugs.

### 3.3 Neurological Disorders

As the research progresses, it is increasingly evident that the homeostasis of gut microbiota is crucial for maintaining brain homeostasis. Any imbalance in the gut microbiota composition and quantity may affect the CNS and the ENS [129]. The communication pathways between the gut microbiota and the brain include metabolic, endocrine, neural, and immune pathways, which work independently or synergistically. The bioactive metabolites produced by a large number of gut microorganisms provide a medium for the gut microbiota to regulate the physiological and pathological processes of the CNS [130]. In this pathway, 90% of the vagal nerve fibers between the brain and the gut are afferent [131], suggesting that the gut often plays the role of a “transmitter” rather than a “receiver.” For example, certain species and genera of gut microbiota can produce dopamine, histamine, γ‐aminobutyric acid (GABA), and serotonin (5‐HT). Those metabolites involved in various functions such as mood regulation and cognitive behavior as neurotransmitters or precursors of neurotransmitters [132]. About 95% of 5‐HT in the human body is produced by enterochromaffin cells in the GI tract, with the remaining approximately 5% found in the brain [133]. In the process of microbiome metabolism, SCFAs produced by the gut microbiota are mainly composed of acetate, propionate, and butyrate, which can exert their effects through G‐protein coupled receptors or histone deacetylases [134] to participate in complex neurological activities. Moreover, there are intricate immune regulatory interactions between the gut microbiota, the gut immune system, and the brain. The metabolites produced by the gut microbiota also regulate the maturation, differentiation, and activation of microglia and astrocytes to mediate various neurophysiological processes. Generally including the maintenance of blood–brain barrier (BBB) integrity, neurodevelopment, neurotransmission, and CNS immune activation [135, 136].

**FIGURE 2:**
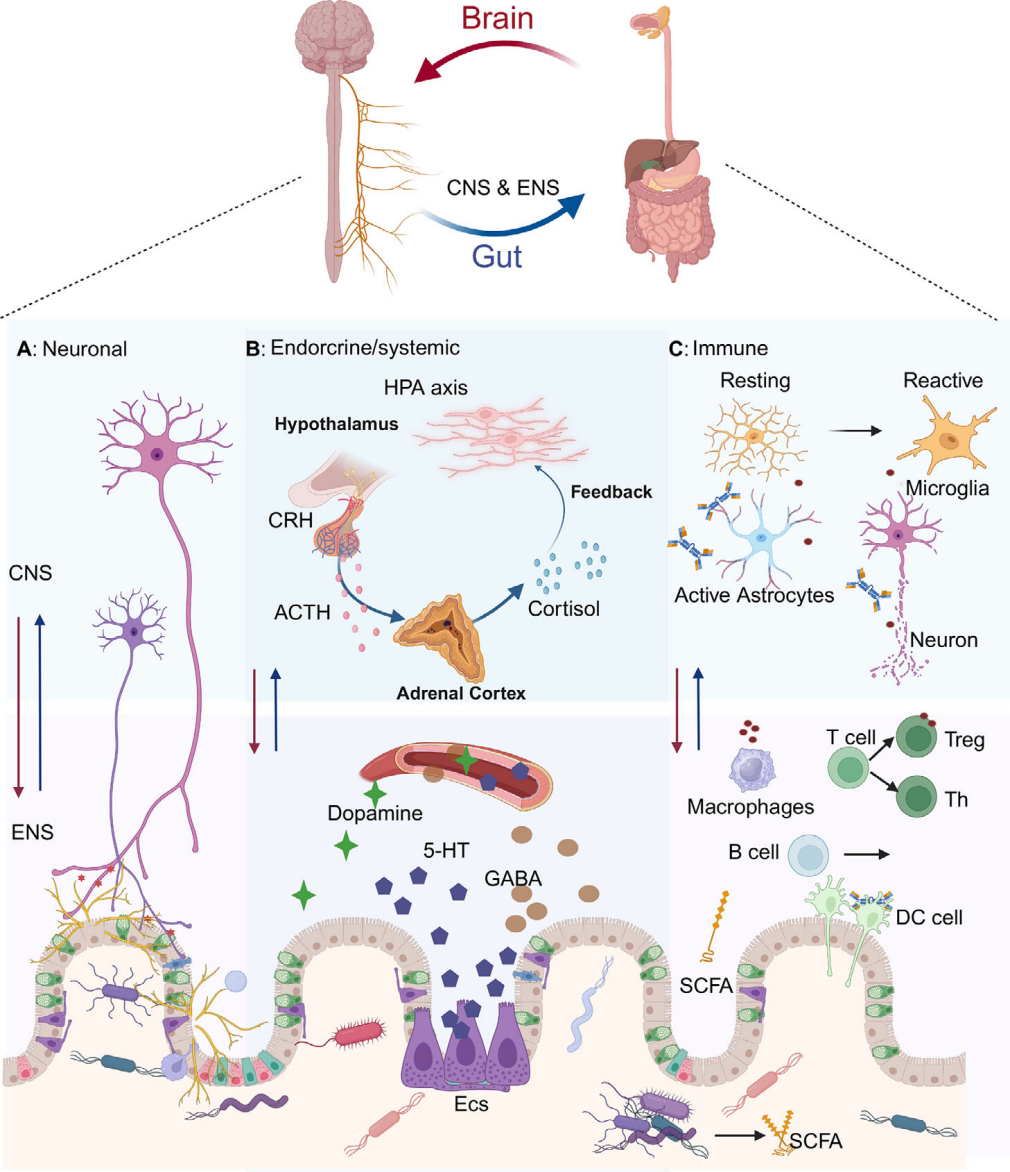
Communication between the gut microbiota and the brain. (A) Signals are transmitted to the enteric nervous system via the vagus nerve and spinal efferent nerves. At the same, gut microbiota components can directly interact with the enteric nervous system and its associated vagal and spinal afferent nerves. (B) Gut microbiota components or metabolites regulate the secretion of neuropeptides. Gut microbiota engage in bidirectional interactions with neuroendocrine signaling pathways mediated by the hypothalamic–pituitary–adrenal (HPA) axis. *Abbreviations*: CRH, corticotropin releasing hormone; ACTH, adrenocorticotropic hormone. (C) The gut microbiota and its metabolites influence immune homeostasis, leading to localized proinflammatory or anti‐inflammatory immune responses. They can also promote the activation of microglia and astrocytes, exacerbating neuroimmune reactions (created in BioRender. Yang, X. (2025) https://BioRender.com/undefined).

With the deepening research on gut microbiota homeostasis, the gut–brain axis has become increasingly clear. There is frequent bidirectional communication and mutual regulation between the GI tract and the CNS through the gut–brain axis. The homeostasis of gut microbiota is closely related to the occurrence and development of many neurodegenerative diseases. However, the specific role and mechanisms of gut microbiota in particular neurodegenerative diseases have not been fully elucidated and require further investigation. High‐quality data generated from preclinical and clinical studies are needed to eventually translate gut microbiota research into clinical practice (Figure 2).

#### 3.3.1 Alzheimer's Disease

Currently, changes in the composition of gut microbiota have been linked to various neurological disorders, although the causal relationship between gut dysbiosis and neurological dysfunction remains elusive. For example, gut microbiota dysbiosis may promote Aβ aggregation, neuroinflammation, oxidative stress, and insulin resistance. All of those promote the onset of AD [137]. AD is a progressive neurodegenerative disorder of the CNS, characterized by a gradual decline in cognitive abilities. The deposition of extracellular Aβ forms amyloid plaques, and the accumulation of intracellular hyperphosphorylated tau forms neurofibrillary tangles remain the primary neuropathological criteria for AD diagnosis. Since *Hardy* and *Higgins* first proposed the amyloid cascade hypothesis in 1992, which suggests that the accumulation of Aβ peptides derived from the amyloid precursor protein is the initial event in the pathogenesis of AD [138]. This hypothesis has remained the dominant theory of AD pathogenesis. In the subsequent long‐term development, an increasing amount of evidence suggests that neuroinflammation is not just a result of AD, but also an important factor in its onset and progression. This has driven research into the role of the microbiome–brain–gut axis in AD. The imbalance of gut microbiota indirectly promotes the development of AD.

The gut microbiota is an important environmental factor for microglia to function in immune and neurological responses throughout the host's life cycle. Evidence suggests that the gut microbiota is essential for the AD pathology and cognitive deficits in AD mouse models [139]. Studies in model animals report that gut dysbiosis may contribute to neuronal damage in AD through Aβ‐dependent mechanisms. For example, in AD mouse models, abnormal accumulation of Aβ in myenteric neurons and activation of innate immunity in the gut precede the onset of CNS neuroinflammation [140]. Similarly, in Tg2576 mice (a transgenic mouse model of AD), gut microbiota dysbiosis, intestinal epithelial barrier dysfunction, and intestinal vascular Aβ deposition occur precede the onset of brain Aβ deposition [141]. Aβ deposits have also been found in the intestinal autopsies of AD patients [141]. All of those suggest that the onset and progression of AD are closely linked to the gut microbiota. SCFAs effectively interfering with Aβ assembly [142], but the derivative metabolite TMAO increases β‐secretase activity, thereby exacerbating the accumulation of Aβ [142]. In addition, the increase in BAs can disrupt tight junctions and increase the permeability of the BBB [143]. It allows more peripheral blood products to enter the CNS. In sum, the imbalance of gut microbiota indirectly promotes the development of AD.

#### 3.3.2 Parkinson's Disease

PD is a chronic neurodegenerative disorder characterized by the loss of dopaminergic neurons in the substantia nigra of the brain. A prominent pathological feature is the abnormal aggregation of α‐synuclein (α‐syn) in CNS. And the death of dopaminergic neurons impairs dopamine production to limit the capacity for neural innervation in PD. The primary dopamine synthesis pathway in the human body involves the phenylalanine–tyrosine–l‐dopa–dopamine pathway, which supplies dopamine to the brain. In this process, tyrosine hydroxylase (TH) acts as a rate‐limiting enzyme, hydroxylating tyrosine to produce l‐dopa in the presence of the cofactor tetrahydrobiopterin (BH4) [144]. And then it converted to dopamine in the brain. Dopamine is a catecholamine neurotransmitter that plays a critical role in motor coordination, as well as in motivation, reward, addiction, learning, and memory. Studies have found that *Enterococcus* species possess abundant TH activity, and improving the gut microbiota can increase dopamine levels in the brain to alleviate PD's symptoms. This is closely associated with l‐dopa produced by gut bacteria entering the brain through circulation and being converted into dopamine [145]. This directly links the gut microbiota to brain function.

Population studies have shown that gut microbiota undergoes continuous changes with the progression of PD [146]. A study involving 490 PD patients revealed the relationship between PD and the gut microbiome through metagenomic analysis. In PD patients, the composition of the gut microbiota is predominantly pathogenic. It is characterized by an increase in pathogens and immunogenic components, dysregulation of neuroactive signals, induction of α‐syn pathological aggregation, and a reduction in anti‐inflammatory and neuroprotective factors [147]. Inflammation is considered a driving factor in the pathogenesis of PD. Modulating the gut microbiota can reduce systemic inflammation by improving intestinal inflammation and gut barrier function. Evidence suggests that FMT can protect against brain local neuroinflammation mediated by the LPS–TLR4 signaling pathway in an MPTP‐induced PD mouse model through the gut–brain axis [148]. In clinical randomized controlled trials, FMT treatment not only improved GI symptoms in PD patients but also effectively alleviated motor dysfunction [149]. Gut microbiota metabolites are also involved in the progression of PD. For example, SCFAs are associated with α‐syn aggregation specific to Thy1–αSyn genotype mice. It pathologically activates microglia, exacerbates neuroinflammation, and promotes motor dysfunction in PD [73]. Studies on PD patients have also demonstrated that low levels of SCFAs are associated with the progression of PD. It is linked to inflammation development supported by SCFAs and disruption of the gut barrier [150, 151, 152]. In addition, free fatty acid receptor 3 (FFAR3)in the ENS also mediates the neuroprotective effects of propionate in PD [153].

As the role of the microbiome–gut–brain axis in PD receives increasing attention, more clinical studies are needed to explore the relationship between gut microbiota changes and the clinical subtypes of PD. Currently, Only little studies have explored differences in gut microbiota among different motor subtypes of PD by performing metagenomic sequencing on fresh stool samples from PD patients [154]. With the development of big data, more technological approaches will available for detecting gut microbiota and their metabolites. Establishing a gut microbiome profile network for PD populations will better contribute to the prevention and treatment of PD.

#### 3.3.3 Autism Spectrum Disorder

With the development of gut microbiota and the gut–brain axis, more interrelations between diseases have come into focus. The prevalence of GI disorders in patients with autism spectrum disorder (ASD) is as high as 70% [155]. These data prompted many studies to link the frequently observed gut microbiota dysbiosis in ASD patients to the regulation of brain function and social behavior. ASD is a group of complex neurodevelopmental disorders characterized by reduced speech, social interaction, and repetitive behaviors with restricted activities. Interestingly, approximately 43–76% patients with ASD have abnormal intestinal permeability [156]. But the incidence of GI symptoms, including abdominal pain, bloating, constipation, and gastroesophageal reflux, is as high as 84% in pediatric patients [157]. It suggest that the microbiome–gut–brain axis may play an important role in the pathology of ASD. The researchers found that when germ‐free mice were colonized with the fecal microbiota from children with ASD, the mice exhibited significant ASD‐like behaviors, demonstrating that the microbiota plays a pathogenic role in ASD [158]. About 20 years ago, the potential link between the microbiota and ASD was proposed. There have been reports that oral vancomycin treatment provided short‐term benefits to a small number of children with ASD [159].

The gut microbiota can influence gene expression and host behavior through various pathways, including the production of neuroactive molecules. For example, the gut microbiota specifically regulates the concentrations of several metabolites in the colon and circulatory system, such as taurine (a metabolite of cysteine or taurocholic acid) and 5AV (a fermentation product of proline) [160]. Taurine is crucial for brain development and can be produced by neurons and astrocytes. 5AV acts as an anticonvulsant in mice and these metabolites mediate communication between the gut and the brain [158]. GABA is an amino acid and the main inhibitory neurotransmitter in the brain. The altered GABA pattern has been identified as a key feature of the neurophysiology in ASD patients [161], while *Bifidobacterium* and *Lactobacillus* are producers of GABA [162]. This prove homeostasis of the gut microbiota plays an important role in the production and maintenance of GABA homeostasis. In addition to changes in intestinal permeability and gut microbiota metabolite‐mediated neuronal activity, immune system defects in ASD patients are associated with alterations in the composition of the gut microbiota. The composition of gut microbiota changes to increase proinflammatory factors such as interferon (INF), IL‐6, IL‐8, and IL‐1b in the brains of ASD patients [163], thereby activating microglia and initiating an immune response. These highlight the important role of the gut microbiome in brain communication, and it may serve as a potential therapeutic target in ASD.

### 3.4 Immune‐Related Disorders

The gut is the region with the highest density of immune cells and the gut microbiota has extensive bidirectional communication with innate immunity [164]. On the one hand, it can significantly influence the development of organized lymphoid structures to affect the activation of both the innate and adaptive immune systems [165, 166]. On the other hand, immune interactions promote the release of inflammatory mediators, including cytokines and chemokines to mediate various physiological processes. Therefore, the dysregulation of the gut microbiota is closely related to various immune‐related diseases.

Dysbiosis of the gut microbiota affects the integrity of the intestinal epithelial barrier, what lead to the entry of antigens (including the microbes or their metabolites) into the bloodstream. These antigens trigger excessive immune responses that cause immune‐related diseases [167]. For example, depletion of the gut microbiota and changes in metabolic activity during the 3‐month period in neonates are associated with childhood multisensitization allergic diseases and T cell differentiation. This is manifested by a significant reduction in the abundance of *Bifidobacterium*, *Akkermansia*, and *Bacteroides*, while the relative abundance of certain fungi, such as *Candida* and *Saccharomyces* is higher [168]. And this may promote CD4^+^T cell dysfunction associated with childhood atopy. In fact, the gut microbiome of allergic children mediated by IgE has common characteristics including an increase in *Firmicutes* and a decrease in Bacteroidetes [169]. In chronic spontaneous urticaria, the gut microbiota exhibits low diversity and SCFAs production, but the level of *Klebsiella pneumoniae* in the gut is elevated. This drive IgE‐mediated inflammatory responses and associated with high disease activity [87].

Besides, the autoimmune diseases are characterized by dysregulated immune responses against self‐antigens, which lead to chronic inflammation. However, alterations in the gut microbiota composition have been observed in various autoimmune diseases, with certain microbial groups being associated with clinical indicators. For example, *Akkermansia muciniphila* is increased in MS [170]. But the relative abundance of the genus *Haemophilus* is decreased in rheumatoid arthritis, which is associated with a reduction in rheumatoid factor titers [171]. Additionally, a decrease in the relative abundance of *Firmicutes* and an increase in *Bacteroidetes* have also been observed in systemic lupus erythematosus patients [172, 173]. The involvement of numerous gut microbiota in the functional and systemic effects on adaptive immune response cells deserve attention [174]. For instance, the cytokine production of macrophages and dendritic cells [175], as well as the induction of Treg cells [174, 176]. Although increasing evidence has identified the role of gut microbiota in these autoimmune diseases, the functional impact of these microbes on diseases remains to be elucidated.

### 3.5 Emerging Associations

The role of the microbiota in health and disease has received extensive research and attention since discovery. Currently, cardiovascular diseases and skin disorders (e.g., psoriasis) are also now considered to be related to body dysfunction caused by dysbiosis. This is a close relationship between the GI and respiratory tracts, as exacerbations of chronic gut and lung diseases are shown to share with disorders of the microbial ecosystem [177, 178]. Furthermore, microbiota or microbial metabolites are also emerging as potential modulators of placenta‐related prenatal diseases.

#### 3.5.1 Cardiovascular Diseases

It is generally believed that the risk factors for cardiovascular diseases include hypertension, atherosclerosis, obesity, diabetes, and so on. However, with the establishment of the network of diseases, it has been discovered that the microbiome plays a role in cardiovascular health [179, 180]. The gut microbiome is involved in the metabolism of choline, phosphatidylcholine, carnitine, and produce TMAO at last. TMAO not only regulates cholesterol balance and BAs levels [181] but also activates the MAPK and NF‐κB signaling pathways [182]. In addition, the common metabolite LPS can induce vascular oxidative stress by activating the TLR4 pathway. This phenomenon induce endothelial dysfunction and vascular inflammation. Apart from this, the gut microbiota can metabolize polysaccharides and proteins into SCFAs, which are closely related to cardiovascular diseases. Specifically, propionate and butyrate have been shown to protect the host from hypertension‐related cardiovascular damage [183, 184]. Additionally, the action of SCFAs on G protein‐coupled receptors [185] further strengthens the role of gut microbiota in blood pressure regulation and cardiovascular diseases progression.

#### 3.5.2 Skin Disorders

Based on the establishment of the gut–skin axis, the important role of microbiota in maintaining skin homeostasis has become clear. When the harmonious relationship between the gut microbiota and the immune system is disrupted, it subsequently affects the skin and promotes the development of skin diseases. For example, the occurrence of atopic dermatitis in infancy is related to the content of arachidonic acid in breast milk. High concentrations of arachidonic acid induce dysbiosis in the infant, significantly increasing the area of skin lesions [89]. Besides, *Faecalibacterium prausnitzii, Akkermansia muciniphila*, and *Ruminoccocus* prevent the colonization of pathogenic bacteria on the skin to prevent psoriasis by competitive inhibition and enhancing the production of SCFAs [186, 187, 188]. Generally, the communication between the gut and skin is established through immune crosstalk, through the immune system cooperating to manage systemic or local inflammatory responses [189]. A good example is that the reduction in the number of potentially beneficial microorganisms in psoriasis disrupt the balance of the immune system. The decrease of *Bacteroides* and *Proteobacteria* exacerbating the occurrence of proinflammatory responses [190]. In sum, a deeper understanding of the potential mechanisms of the gut–skin axis and the connection between the gut and skin diseases can provide guidance for healthy skin management and the establishment of a healthy gut microbiota. It also will to assist in the search for drugs related to the treatment of skin diseases.

#### 3.5.3 Respiratory Diseases

The gut–lung axis is a complex system that connects, modifies, and influences the microbiota from the GI tract to the lungs. Bacterial products of the gut microbiota may cross the epithelial barrier into the blood and regulate the gut–lung axis [191]. The lung and intestine demonstrate a bidirectional relationship, with a hallmark characteristic of interconnected immune and inflammatory regulatory networks. On the one hand, soluble components or metabolites of gut microbiota influence lung diseases through immune regulation. As a key component of SCFAs produced by gut microbiota, butyrate has been shown to exert significant effects on a range of pulmonary diseases, such as allergic asthma, chronic obstructive pulmonary disease (COPD), and pulmonary fibrosis [192, 193]. These metabolites are capable of activating GPCRs (HCAR2/GPR109a, FFAR2/GPR43, and FFAR3/GPR41) located on the intestinal epithelial cell surface or undergoing internalization via cellular transporters. SCFAs can be utilized for ATP generation in the mitochondria, act as HDAC inhibitors in the nucleus, or be transported out of the cell and into the lamina propria, subsequently entering the bloodstream. Therefore, SCFAs can regulate the functions of various target tissues, including the lungs, kidneys, and brain. At the same time, it can regulate immune cells, inducing lymphocyte tolerance and participating in inflammatory regulatory networks [194].

On the other hand, lung and gut microbes interact by altering the immune system. Both intestinal and airway mucosa express common homing chemokine receptors, such as chemokine ligand 28 (CCL28), which mediate lymphocyte migration [195]. Gut‐associated lymphoid tissue is an important link between the lungs and intestines, playing a key role in inducing immunity and controlling communication between the intestinal mucosa and systemic immunity [196]. The mucosal surfaces of the lungs and intestines are rich in group 2 innate lymphoid cells (ILC2s). ILC2s mature through the lung‐gut axis to acquire normal functions. Developmental defects of ILC2s in the lungs significantly affect the number and function of ILC2s in the intestine [197]. Allergens that promote asthma increase the number of ILC2s in the lungs and intestines, indirectly proving the correlation between the lungs and the intestines. Clinical studies also indicate that asthma and UC are associated with IL‐33 signaling‐mediated intestinal inflammation, particularly in individuals under the age of 16 years [198]. NF‐κB inflammatory signaling disrupts lung fibrosis caused by gut–lung microbiota dysbiosis in the context of diabetes by supporting mucosal immune crosstalk [199]. The interactions between the gut microbiota and its metabolites with the immune system promote the development of pulmonary diseases. The establishment of bidirectional communication between the gut–microbiota–lung axis has enhanced the understanding of treatments for pulmonary diseases. For example, targeting the gut microbiome can significantly improve acute lung injury caused by LPS [200]. Probiotic supplementation can improve lung function and has a beneficial effect on COPD [201]. Additionally, specific microbiomes have been found to effectively predict the responsiveness of patients to immunotherapy in lung cancer patients [202]. And the survival of non‐small cell lung cancer patients is associated with gut bacterial diversity [191]. The development of the gut–lung axis has deepened our understanding of respiratory diseases. Apart from this, the interaction between the microbiome and cancer treatment has opened new avenues for improving efficacy and reducing side effects.

#### 3.5.4 Placental‐Origin Diseases

The maternal microbiome is a critical regulator of health during pregnancy and has a significant impact on offspring development [203, 204]. Microbiota or microbial metabolites are emerging as potential modulators of placenta‐related prenatal diseases, such as fetal growth restriction, preeclampsia, and preterm birth. The mother and fetus establish extensive connections through the highly vascularized placenta. The placental labyrinth consists of maternal and fetal blood spaces, separated by trophoblast cells, the basement membrane and fetal endothelial cells. Together, these structures mediate gas and nutrient exchange to sustain fetal growth and development [205]. Therefore, the maternal gut microbiota regulates not only the metabolites within the mother but also those of the fetus itself. Studies have found that the functional metabolites produced by the maternal gut microbiota during pregnancy are critical for supporting placental growth and angiogenesis in mice [206]. Moreover, supplementation with SCFAs during pregnancy can prevent placental growth restriction and vascular dysfunction in maternal malnutrition model [206]. In an obese pig model, a reduction in maternal gut microbiota diversity was also shown to lead to decreased SCFAs, inducing placental oxidative damage and mitochondrial dysfunction [207]. At the same time, the placenta mediates the SCFAs transfer from maternal circulation to fetal circulation to promote fetal neurodevelopment [208]. In a sheep model, gut microbiota dysbiosis caused by maternal environmental pollutants promotes placental cell apoptosis in pregnant ewes through the gut–placenta axis. It leads to fetal growth restriction [209]. The impact of environmental pollutants on pregnants and fetuses is often closely related to the gut–placenta axis. Population studies show that microplastics cause maternal gut microbiota dysbiosis and enter the placenta to disrupt offspring development [210]. Reduced gut microbiota diversity in children aged 4–6 years is often associated with prenatal exposure to environmental pollutants and their accumulation in the placenta [211]. Tracing back to early life, intrauterine fetal growth and development heavily depend on adequate placental function. Poor maternal gut microbiota is associated with impaired placental homeostasis and fetal development. The most direct evidence is that maternal *Bifidobacterium* promotes placental morphogenesis and regulates fetal growth through the gut–placenta axis [212]. Moreover, maternal gal‐3 deficiency‐induced gut microbiota dysbiosis leads to fetal growth restriction too [213]. It is clear that changes in the maternal gut microbiota and metabolites may lead to impaired placental adaptation. And changes in the gut–placenta axis can cause developmental alterations in the offspring [214].

**FIGURE 3:**
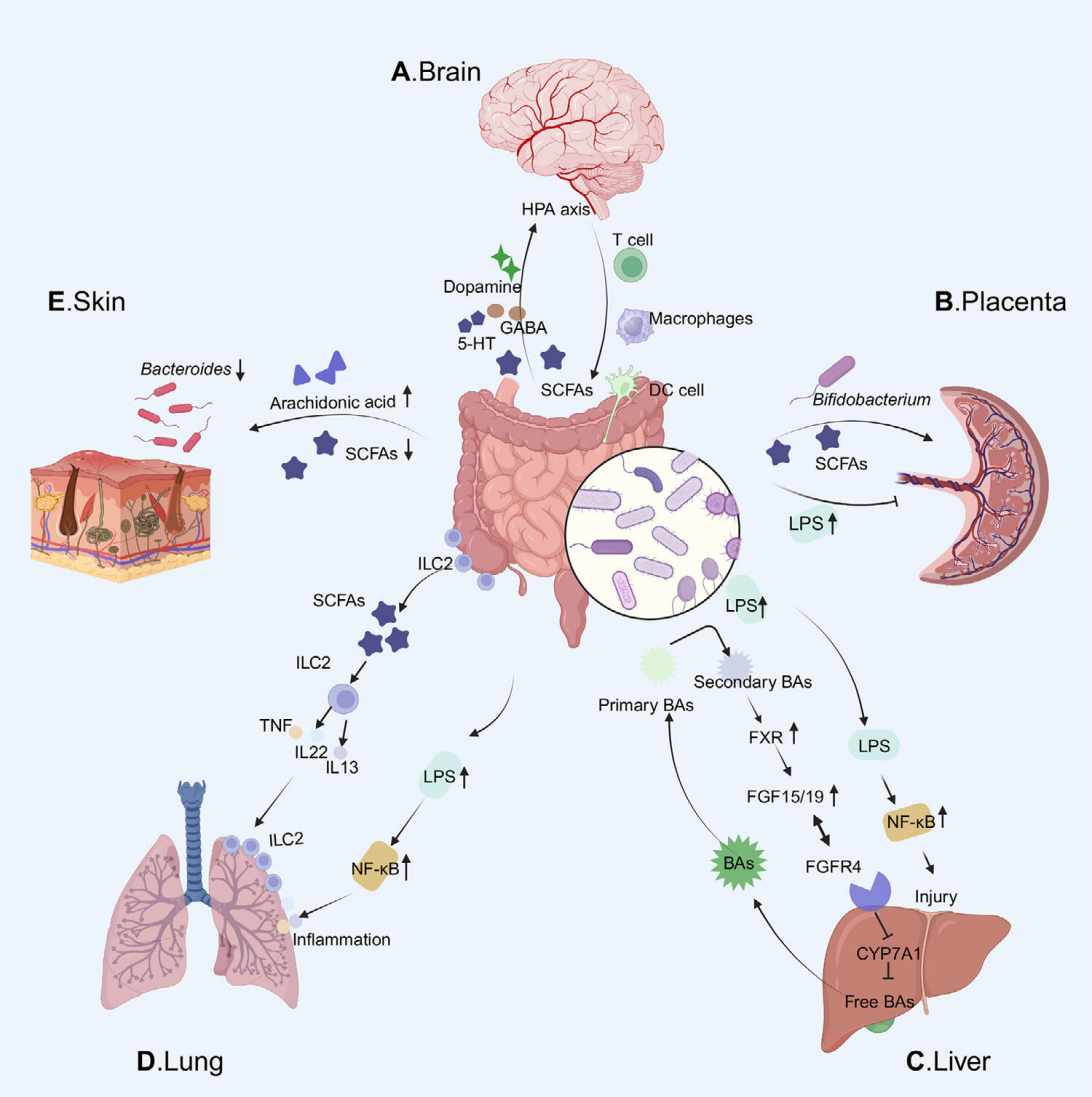
Communication between the gut microbiota and other organs. (A) The gut microbiota and the brain engage in bidirectional communication through various metabolites (SCFAs, 5‐HT, GABA, dopamine), endocrine pathways (HPA axis), and immune pathways. (B) Microbiota dysbiosis or microbial metabolites are emerging as potential regulators of placenta‐related prenatal diseases. Certain beneficial bacteria, such as Bifidobacterium can promote placental morphogenesis and microbiota metabolites like SCFAs can prevent placental growth restriction and promote placental angiogenesis. However, excessive LPS cause fetal growth restriction through the gut–placenta axis. (C) The liver secretes BAs into the intestine via the biliary system, where they are reabsorbed. BAs metabolism in the gut–liver axis is primarily regulated by FXR, with secondary BAs promoting FXR expression. In turn, it inhibits BAs production and influx, facilitates BAs efflux, to alleviates excessive BA accumulation in the liver. However, gut microbiota dysbiosis can lead to systemic microbial translocation into the hepatic portal circulation. Increased LPS activates the NF‐κB signaling pathway to exacerbate the progression of liver diseases. *Abbreviations*: FGF15/19, fibroblast growth factor15/19; FGFR4, fibroblast growth factor receptor4; CYP7A1, cholesterol 7‐alpha hydroxylase; NF‐κB, nuclear factor‐κB. (D) The crosstalk within the gut–lung axis is closely linked to the circulatory and lymphatic systems. Beneficial microbiota produce metabolites such as SCFAs can enhance the activation of innate lymphoid cells (ILCs), forming a natural protective barrier. Conversely, dysbiosis‐induced increases in LPS trigger the NF‐κB signaling pathway, leading to inflammatory infiltration and lung function impairment. (E) Microbiota dysbiosis induce excessive arachidonic acid and reduce SCFAs wl promote the development of skin diseases. And the reduction of beneficial microbes such as Bacteroides exacerbates proinflammatory responses (created in BioRender. Yang, X. (2025) https://BioRender.com/undefined).

With the development of genomic technologies such as metagenomics, metatranscriptomics, and metaproteomics, it may be possible to predict the characteristics of the placenta‐associated microbiome [215, 216]. It could serve as therapeutic targets for placenta‐related diseases. With the rapid development of big data, it may be possible to construct gut–placenta data networks across different ethnic backgrounds. This work will contribute to fully understand the importance of host–microbiome symbiosis during pregnancy. Combine AI to predict microbiome‐related target changes, the drugs and probiotics that regulate the balance of the gut microbiota to modulate the gut–placenta axis may become new candidate approaches for alleviating gut‐derived placental damage or fetal growth restriction (Figure 3).

## 4 Prevention of Gut Microbiota Dysbiosis

The prevention strategies for gut microbiota dysbiosis include multiple interventions that work together to maintain the balance of the gut microecology. Lifestyle and diet are the fundamental preventive measures. Through a balanced diet, increased intake of dietary fibers, and improved daily habits to promote the growth of beneficial microbes. Additionally, avoiding unnecessary antibiotic use helps protect the diversity of the gut microbiota. These different strategies are interconnected and work together to support the maintenance of a healthy gut.

### 4.1 Lifestyle and Dietary Interventions

The maintenance of gut microbiota homeostasis is related to daily life. According to TCM, disease goes in by the mouth. Diet plays a crucial role in maintaining the balance of the gut microbiome and is one of the key factors that directly influence the human microbiota healthy. In the process of urbanization, factors associated with urbanization diets can induce imbalances in the structure and composition of the gut microbiota. For example, the large amounts of red meat consumed in urban diets are metabolized by the gut microbiota into hydrogen sulfide and trimethylamine. And then, these metabolites are converted by the liver into TMAO, which promote the development of atherosclerosis and weaken the intestinal barrier [217]. Among various dietary components, dietary fiber has the closest relationship with the gut microbiota. It contains multiple microbiota‐accessible carbohydrates that cannot be digested by the human but can directly provide nutrition for the gut microbiota. Long‐term low dietary fiber intake disrupts the colonic mucus barrier, increasing susceptibility to pathogens [218]. Therefore, the metabolic dynamic balance between dietary fiber and the syntrophic microbiota shapes the gut microbiota homeostasis. The extinction of gut microbiota with low abundance of *Bacteroidete*s caused by a low‐fiber diet in mice was irreversible [219], directly underscores the importance of a balanced diet. Besides, the intake of fermented food in moderation is beneficial for gut health. It provides the probiotics, prebiotics, and derived bioactive substances to maintain the stability of the gut microbiota [1]. Polyphenols have antioxidant and anti‐inflammatory activities and can be metabolized by gut microbiota into active phenolic compounds. Studies have found that polyphenol‐rich diet may help enhance the gut barrier function by increasing specific metabolites, such as butyrate [220, 221]. In daily life, a balanced diet is the most direct and effective way to prevent gut microbiota imbalance. A high‐fiber intake promotes the growth of beneficial bacteria and inhibits the proliferation of harmful bacteria to maintain a balanced gut microbiome. In addition, moderate intake of prebiotic substances such as resistant starch and oligosaccharides can provide an energy source for beneficial bacteria and enhance their ability to colonize the gut. A reasonable intake of nutrients such as protein and fat help to regulate the structure of the gut microbiota and reduce the incidence of dysbiosis. Finally, reducing unhealthy dietary habits such as high‐sugar, high fat, and processed foods reduce the growth potential of harmful bacteria in the gut, thereby promoting a healthy gut microbiome homeostasis.

### 4.2 Avoiding Unnecessary Antibiotic Use

Antibiotics are the most used drugs for treating infectious diseases. They target not only pathogenic bacteria but also beneficial induce changes in gut microbiome homeostasis [222]. Antibiotics disrupt the intestinal barrier, leading to a reduction in gut microbiota diversity. And it will change the microbial abundance and alterations in intestinal metabolites [223]. These effects may have short‐term or long‐term impacts on the gut microbiota and can affect multiple systems [224]. For instance, exposure to antibiotics is associated with an increased risk of developing IBD. Compared with patients who have not used antibiotics, those who have received three or more times of antibiotics have a 55% higher risk of IBD [225]. In addition, a study found that early administration of antibiotics reduced indole‐3‐propionic acid, what induce gut microbiota dysbiosis and metabolic abnormalities. It increased the susceptibility to allergic airway inflammation in adulthood [226]. The another, the acute loss of oxalate microbial metabolism in the gut microbiota caused by antibiotic significantly increases the incidence of urinary tract stone disease [227]. Excessive antibiotic causes changes in the gut microbiota and metabolism what lead to cognitive dysfunction through the gut microbiome–brain axis [228, 229]. Moreover, the gut and liver are physiologically closely related through “gut–liver axis.” Antibiotic‐induced gut microbiota dysbiosis significantly affects gene expression both in the gut and liver. FMT has a restorative effect on the genes of the gut and liver [230]. Based on population studies report that the use of antibiotics can alter the human gut microbiota, and it may take months or even years for the original composition of species to be restored. Through animal experiments, it was verified that the diversity of the gut microbiota and beneficial bacteria were reduced after exposing mice to ceftriaxone. This phenomenon persisted for up to 14 months [231]. Therefore, avoiding unnecessary antibiotic use plays a crucial role in maintaining the homeostasis of the gut microbiota. This is an useful way to reduce the adverse impact on health.

### 4.3 Early‐Life Interventions

The establishment of the gut microbiota in early life has a lasting impact on subsequent health. The initial colonization of the GI tract by gut microbiota is thought to begin at birth. The infant is exposed to maternal microbiota from mother and other environmental factors including skin, vagina, feces, and breastfeeding [232]. It is noteworthy that the gut microbiota of infants born vaginally is very similar to the microbial composition of the mother's vagina. But newborns delivered by cesarean section are enriched with microbes from the human skin and the surrounding environment [233]. Based on the characteristics of vertical transmission of the gut microbiota, the health of the mother directly determines the establishment of a healthy microbial community in early life. And on the other hand, breast milk microbiota can directly seed the infant's gut microbiota. Moreover, the effect of breast milk on the infant's gut microbiota is dose dependent [234]. The difference in the log‐ratio of relative abundance of gut bacterial taxa between formula‐fed and breastfed infants shows significant heterogeneity. The longer the duration of exclusive breastfeeding, the less gut microbiota dysbiosis related to diarrhea. Generally, the unique microenvironment of each intestinal region selects for the growth of specific microbiota, with the distal intestine being the primary habitat for the intestinal microbiota [235]. *Firmicutes* and *Bacteroidetes* are the most abundant phyla in the human gut microbiota [236]. In the first 6 months of life, the gut microbiota diversity, microbiome age, relative abundance of *Bacteroidetes* and *Firmicutes*, and the predicted microbial pathways associated with carbohydrate metabolism were consistently higher in formula‐fed infants. But the relative abundance of pathways related to lipid metabolism, vitamin metabolism, and detoxification were lower [237]. Another way, the number of *Proteus* species in formula‐fed infants is significantly lower than in breastfed infants after cesarean section. This suggest that when *Bacteroides* are depleted in the gut microbiota (a typical feature in the early life of cesarean‐born infants), formula feeding further depletes *Proteus* species [238]. This phenomenon increases the risk of adverse health outcomes in cesarean‐born infants [239]. Regardless of the mode of delivery, healthy breastfeeding plays a consistent role in maintaining the development of the infant's gut microbiome homeostasis. It promotes the early colonization of healthy gut microbiota and the establishment of microbial communities. Therefore, focusing on maternal health is the first step in establishing a healthy microbial homeostasis in the early stages of life. It is the foundation for preventing adverse life outcomes caused by gut dysbiosis.

## 5 Therapeutic Approaches Targeting Dysbiosis

The treatment methods for gut microbiota dysbiosis aim to improve health by restoring the balance of the microbiota. As the understanding of gut microecology deepens, the restoration of the gut microbiota is no longer reliant on altering a single factor, but rather involves a multifaceted process that regulates various mechanisms. By precisely intervening in the composition and function of the gut microbiota, it is expected that diseases caused by dysbiosis can be effectively alleviated. Next, we will explore in detail the specific mechanisms and applications of these different approaches.

### 5.1 Probiotics and Prebiotics

**FIGURE 4:**
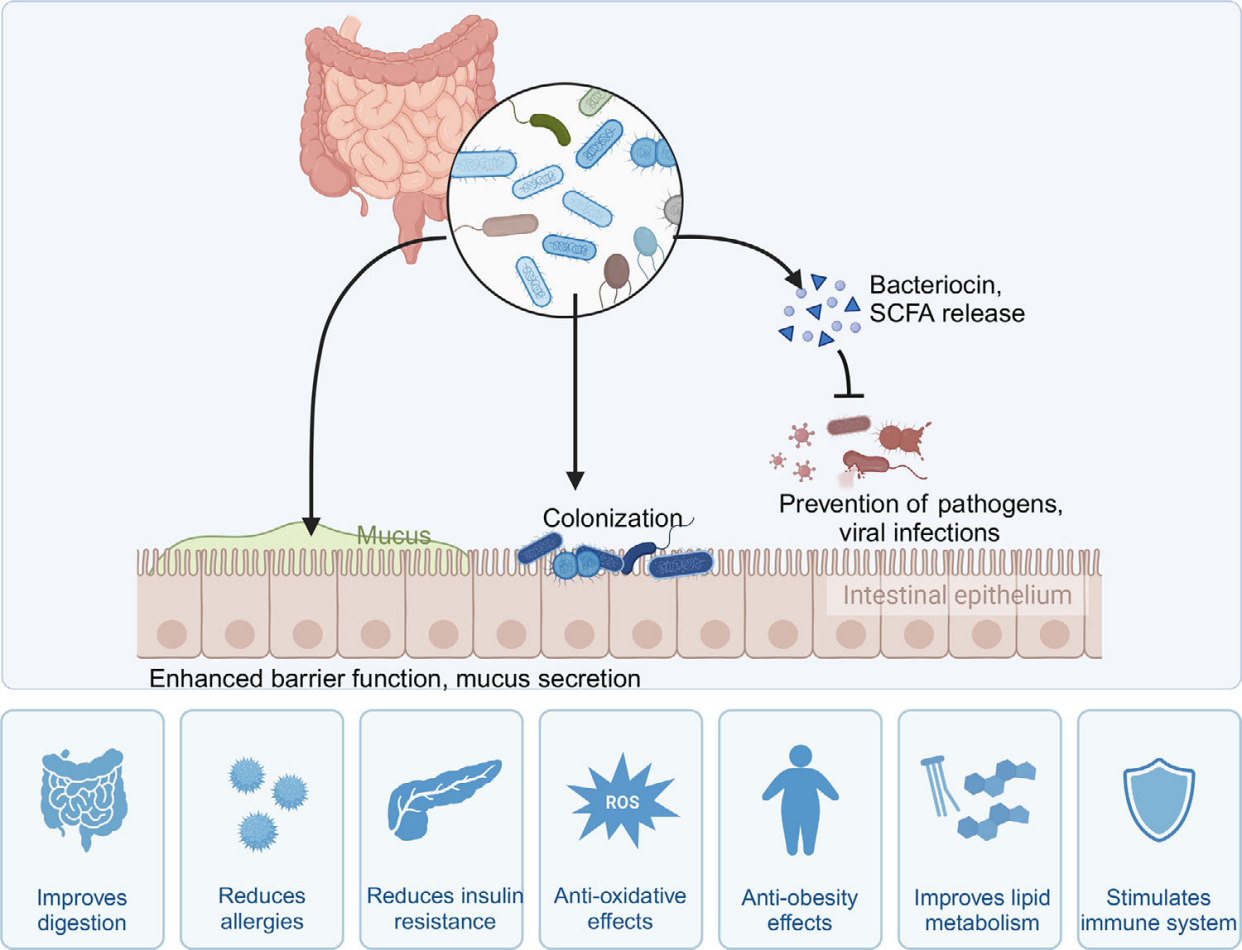
Health benefits of probiotics. Exogenous therapeutic probiotic supplementation achieves favorable therapeutic outcomes through three primary pathways: inhibition of bacterial pathogen growth and repair of the intestinal barrier; long‐term supplementation with exogenous probiotics reshapes the gut microbiota by competing for ecological niches. This competition include compete for nutritional substrates to restore intestinal microecology by influencing microbial composition; probiotics promote the host's synthesis of essential nutrients such as amino acids and vitamins to increase the production of SCFAs (created in BioRender. Yang, X. (2025) https://BioRender.com/undefined).

The International Society for Probiotics and Prebiotics defines probiotics as live microorganisms that can confer health benefits to the host when administered in adequate amounts. It not only produce anti‐inflammatory metabolites to downregulate inflammatory factors, but also help inhibit pathogen growth, repair the gut barrier, and regulate the proliferation and differentiation of immune cells [240]. Probiotics promote the synthesis of nutrients such as amino acids and vitamins in the host. And it increase the levels of SCFAs and lower pH of the gut environment to inhibit the growth of pathogens [241]. Probiotics typically do not colonize in the intestines. Long‐term exogenous supplementation of probiotics can influence the microbial composition and reshape the gut microbiota. By ecological niche competition, including competition for nutritional substrates to alleviate disease symptoms. Prebiotics refer to substrates that are selectively utilized by microorganisms to confer health benefits. For example, the inulin and fructooligosaccharides can be selectively fermented by gut microbiota. Prebiotics can regulate gut microbiota and immune responses by stimulating microbial metabolism. Compared with use single or multistrain probiotics, using prebiotics for intervention has the potential to improve the gut microbiota status more comprehensively. In a cohort study of healthy individuals, it was shown that the intake of prebiotics (galactooligosaccharides) can reduce cortisol responses during wakefulness and improve mood bias [242]. It also directly demonstrated the regulatory effect of prebiotic supplementation on the microbiota–gut–brain axis. Moreover, prebiotics do not involve the introduction of microorganisms, have a low incidence of adverse events, and possess a broader accepted safety profile (Figure 4).

The homeostasis of the gut microbiota plays a crucial role in maintaining health and delaying the development of various diseases. In recent years, researchers have broadly summarized the mechanisms of probiotic therapy effectiveness as the “additive and subtractive” effects [243]. The additive effect includes the restoration and maintenance of gut balance through substances like SCFAs produced by probiotics, as well as enhancing intestinal mucosal barrier function by promoting the production of tight junction proteins and mucins. The other way, the subtractive effect includes various mechanisms such as the breakdown of harmful substances, reduction of inflammation levels, and competition with pathogens for resources, all of which work to neutralize adverse effects through the metabolism and degradation of harmful substances [244]. There may be strain‐specific effects in different diseases. For example, the supplementation of specific strains of *Lactobacillus* and *Bifidobacterium* in cardiovascular diseases helps lower cholesterol levels by breaking down BAs and binding cholesterol [245]. *AbfA* cluster *Bifidobacterium longum* preferentially enhances arabinan utilization and is more beneficial for relieving constipation [246]. As substrates, prebiotics function more by providing competitive growth advantages and indirectly affecting the overall composition of the intestinal flora. Changes in dominant bacteria directly affect the production of metabolites beneficial to gut health and overall function. For example, supplementation with oligofructose‐rich inulin in adults with T1DM can significantly reduce C‐peptide levels and increase the relative abundance of the beneficial bacterium Bifidobacterium [247]. Changes in Bifidobacterium abundance lead to alterations in SCFAs and strengthen the intestinal barrier. Currently, clinical research related to probiotics and prebiotics is developing rapidly. The application prospects of probiotics and prebiotics are full of potential, but they also face many challenges. How to ensure the effectiveness of different probiotics or prebiotics, the accumulation of clinical trial data, and the realization of personalized treatment all require further in‐depth research (Table 2).

**TABLE 2:** Evidence from animal and human studies for probiotics and prebiotics.

Disease models	Treatment	Goal/mechanism	References
**Animal studies**			
AD	Prebiotics (docosahexaenoic acid, DHA)	Counteracting tau hyperphosphorylation and cognitive loss	[248]
CRC	Prebiotics encapsulate probiotic spores	Produces anticancer short‐chain fatty acids (SCFA)	[249]
CRC	Prebiotic nanoparticles	Promote the proliferation of probiotics and the production of short‐chain fatty acids, antitumor	[250]
AD	Prebiotics (DHA)	Increase synapse formation	[251]
Diarrhea and hypercholesterolemia	Probiotics (*Lactobacillus*)	Alteration of bile acid composition and signaling by BSH activity of probiotic strains	[252]
NAFLD	Probiotics (Lactobacillus)	Inhibits the synthesis of endogenous cholesterol and promotes the secretion of cholesterol into bile acid through the intestinal FXR–FGF15 signaling pathway	[253]
Psoriasis	Probiotics (*Lactobacillus pentosus* GMNL‐77)	Significantly reduced the production of proinflammatory cytokines in the skin, T cells of IL‐17 and IL‐22 in the spleen, and the area of ​​erythematous desquamative lesions	[254]
Arthritis	Long‐chain inulin‐type fructans and short‐chain inulin fraction fructo‐oligosaccharides	Reduce the incidence of arthritis and colitis	[255]

Disease	Treatment	Goal/mechanism	Clinical information	Reference
**Human studies**				
T2DM	Prebiotics (resistant dextrin)	Improve immune system function and promote SCFA production	Iranian registry (IRCT201110293253N4)	[256]
IBS	Prebiotics (short‐chain fructo‐oligosaccharides)	Stimulates the growth of *Bifidobacterium*	NCT00806104	[257]
ASD	Prebiotics (galacto‐oligosaccharides)	The abundance of *Lachnospiraceae* increased significantly, and fecal and urine metabolites changed significantly	NCT02720900	[258]
ASD	Prebiotics (galacto‐oligosaccharides)	Increased abundance of *Bifidobacterium* and increased acetate and butyrate fatty acids	Not applicable	[259]
Depression	Probiotics	Significantly reduced depression scale scores	Not applicable	[260]
ASD	Probiotics (*Lactobacillus acidophilus*)	Anxiety and communication problems improved	NN204316234	[261]
ASD	Probiotics (*Lactobacillus acidophilus, Lactobacillus rhamnosus*, and *Bifidobacterium longum*)	Gastrointestinal problems improved and ATEC scores decreased	Trial registration number: UMIN‐CTR Study Design: Trial Number UMIN000026157	[262]
ASD	Probiotics	Reduced the abundance of *Desulfovibrio* and *Bifidobacterium* and significantly decreased the level of TNFα	Not applicable	[263]
Psoriasis	Probiotics	Remodeling the gut microbiome	The Research Ethics Committee of the Hong Kong Doctors Union (protocol number HKSGM‐2020AD‐Study‐protocol‐vl‐20220211)	[264]
Depression	Probiotics (*Bifidobacterium*)	Regulated intestinal microbiome and tryptophan metabolism to alleviate major depression	Chinese Clinical Trial Registry (No. ChiCTR2100046321	[265]
Obesity	Probiotics (*Akkermansia muciniphila*)	Reduced insulinemia and plasma total cholesterol, improved insulin sensitivity	NCT02637115	[266]
CRC	Probiotics (*B. infants*, *L. acidophilus*, *E. faecalis*, and *B. cereus* tablets )	Significantly reduce the chemotherapy‐induced complications, including gastrointestinal symptoms such as abdominal distension, abdominal pain, constipation, diarrhea, and intestinal flora imbalance.	Chinese Clinical Trial Registry (No. ChiCTR2000040916	[267]
ASD	Prebiotics (fructo‐oligosaccharide), probiotics	Increase the beneficial bacteria (*Bifidobacteriales and B. longum*) and suppression of suspected pathogenic bacteria (*Clostridium*), significant reduction in the severity of autism and gastrointestinal symptoms	Chinese Clinical Trial Registry (Chictr.org.cn, ChiCTR2000028985	[268]
Caries	Prebiotics, probiotics	Reduce the occurrence of dental caries in children	NCT03928587	[269]
Depression	Prebiotics, probiotics, synbiotics	Appropriate benefits	Not applicable	[270]
Psoriasis	Probiotics, precision prebiotic oligosaccharide mixture	Anti‐inflammatory, regulating cytokine activity, improving quality of life	The Ethical Commission of the “Iuliu Haţieganu” University of Medicine and Pharmacy Cluj‐Napoca (No. 267/06.30.2021)	[271]
Obesity	Probiotics (*Bifidobacterium*), prebiotics (galacto‐oligosaccharides)	Both can improve the intestinal barrier function of obese adults when used alone, but have no synergistic effect when used as synbiotics	NCT02355210	[272]
Obesity	Synbiotics (*Bifidobacterium and Lactobacillus acidophilus* strains, prebiotic ingredient is a mixture of trans‐galacto‐oligosaccharides)	Normalize blood sugar and increase the abundance of *Lactobacillus*	NCT03123510	[273]
NFLD	Synbiotics (fructooligosaccharides + *Bifidobacterium animalis subsp. lactis*)	Effective in improving intestinal flora, but ineffective for liver fibrosis markers	NCT01680640	[274]
T2DM	Synbiotics (*Lactobacillus paracasei Shirota* strain and *Bifidobacterium breve Yakult* strain, and galacto‐oligosaccharides)	Improved intestinal environment in T2DM patients, with an increase in the number of two Lactobacillus species (especially *Lactobacillus* and *Lactobacillus*)	The University Hospital Medical Information Network Clinical Trials Registry, a nonprofit organization in Japan, (UMIN000032057, registration date: 2 April 2018)	[275]
Irritable bowel syndrome with diarrhea	Synbiotics (*Lactobacillus and Bifidobacterium*, and short‐chain fructo‐oligosaccharides)	Improvements in IBS‐SSS scores related to flatulence and bowel habits, as well as overall IBS symptoms	NCT04206410	[276]

### 5.2 Fecal Microbiota Transplantation

FMT refers to the process of transferring a donor's fecal solution into the recipient's GI tract to treat diseases. The most prominent achievement of FMT in disease treatment is to cure recurrent *Clostridioides difficile* infections [277], with a cure rate up to 90% [278]. It has been proven to be a reliable therapeutic alternative, serving as a substitute for vancomycin [279, 280] and fidaxomicin [281]. Subsequently, FMT has also been reported to be applied in diseases such as cancer [282, 283], MS [284], PD [149, 285], NAFLD [286], diabetes [287], graft‐versus‐host disease [288], and progeria [289]. Although promising results have been shown, most studies have been conducted in model animals or have limitations in sample size. The further extensive research is still needed. The current exploration of the therapeutic mechanisms of FMT includes the following aspects: (1) FMT accelerates the decolonization of multidrug‐resistant bacteria through direct microbial replacement and prevents the recurrence of multidrug resistance [290]; (2) FMT reintroduces essential metabolic products beneficial to host recovery, including SCFAs, BAs, antimicrobial peptides, and so on. And then improve the microbiota–gut–brain axis [148]; (3) the recolonization of bacteria after direct ecological competition helps restore immune function and reduces the damage to the host caused by abnormal gut microbiota colonization [291, 292]. The currently advancing FMT treatment is largely safe and effective, but it still faces many challenges. For example, feces are composed of a series of heterogeneous microorganisms, including bacteria, viruses, fungi, parasites, as well as human cells, mucus, and metabolites. These components are closely related to the donor's factors, such as age, lifestyle, and medication use [293]. Therefore, feces cannot be easily compared with standardized, reproducible microbial mixtures. All the uncontrollable factors far outweigh those in any commercial formulation. To promote the treatment of FMT, ensuring standardization, reproducibility, and high precision of the treatment is a major challenge.

Donor selection is the most challenging step in FMT, with its multilayered complexity. It is essential to ensure the safety of the transplanted material and prevent the transmission of infectious pathogens. FMT is likely to become a double‐edged sword. In order to keep the long‐term safety of FMT, future research should make full use of the development of big data to screen and assess microbial risks through metagenomic strain tracking. However, for the recipient, the gut ecosystem of the beneficiary itself is a complex niche and shaped by both host and environmental factors. How the stable state is restored after acute interference of FMT is the result of the interaction of multiple factors, including genetic immune factors, environmental factors, antibiotic use, and other crosstalk interactions [294]. Therefore, the advancement and application of FMT still lack a complete and controllable process from microbiome to clinic. Efforts must still be devoted to developing other microbiome‐based therapies that can be implemented after FMT. For example, novel live biotherapeutic products composed of synthetic microbiomes, or specific combinations of metabolites and microbiomes that can be identified and generated in a highly controlled and reproducible manner.

### 5.3 Postbiotics and Synbiotics

Synbiotics are a combination of probiotics and prebiotics, where prebiotics enhance the activity of probiotics, provide a source of fermentable fibers, and act as general prebiotics. Synbiotics are divided into two categories: synergistic synbiotics and complementary synbiotics. In synergistic synbiotics, the substrate is selectively utilized by the coactive microorganisms, while complementary synbiotics mainly target the host microorganisms. In neurodegenerative diseases, the use of synbiotics has shown therapeutic efficacy. Long‐term supplementation of synbiotics in clinical AD patients reduced peripheral oxidative stress and inflammation levels, and had a beneficial effect on improving cognitive function [295]. In PD, compared with probiotics, synbiotics have also been observed to have a small degree of therapeutic effect and their treatment seems to have received positive feedback [296]. However, not all treatments with probiotics are effective. In studies involving patients with atopic dermatitis, significant benefits were not fully observed [297], which may be related to the combination of probiotics used.

Postbiotics refer to beneficial dead microorganisms or their components that confer health benefits to the host, including nonliving entities such as SCFAs, secondary BAs, and so on. Postbiotics are considered the evolution of probiotics with higher safety, especially for immunocompromised patients and infants. The standardization of postbiotics is easier [298], and with a longer shelf life making their application potentially more widespread. Currently, a lot of research focuses on SCFAs as key postbiotics, including propionate, butyrate, acetate, and valerate, for disease treatment [299, 300, 301, 302]. The impact of postbiotics on the microbiota is temporary, but it can provide protective modulation against pathogens and regulate the host's existing beneficial bacterial strains. All of them support the maintenance of host homeostasis. Second, certain progenitors can enhance the mucosal barrier function [303] and regulate immune responses by altering the secretion of proteins. In immune regulation, TLRs are the most common targets in ligand‐based drug discovery strategies, making postbiotics products potential candidates for inflammatory and autoimmune diseases [304]. The correct use of prebiotics, postbiotics, and other derivative products of probiotics holds development potential for the regulation of gut microbiota or its metabolic activity [305]. For these products, we need to achieve the purification of their components, standardization of production, and consistency in quantifying effects to create highly specialized and safe products ultimately. It is essential for personalized treatment based on patient characteristics and is necessary for the future of precision medicine.

### 5.4 Traditional Chinese Medicine

TCM is plant material with medical value extracted and prepared from natural plants. TCM primarily serves as an important oral medication and has a close interaction with the gut microbiota. Existing studies have shown that TCM restores the homeostasis of the gut microbiota by influencing the gut microbial community [306]. Shenling Baizhu San can regulate inflammatory factors and increase the number of SCFA‐producing bacteria (*Prevotella* and *Oscillospira*) [307, 308]. Ganshuang granules alleviate liver fibrosis by restoring intestinal permeability, increasing the abundance of *Bacteroidetes*, and balancing intestinal dysbiosis [309]. In recent years, the use of probiotics to ferment TCM has become a research hotspot. Compared with TCM, probiotic‐fermented Chinese medicine has the advantages of enhancing efficacy, reducing side effects, and significantly improving the gut microbiome [310]. Research has found that Fermented Yupingfeng polysaccharides significantly increase microbial diversity and the abundance of cellulolytic bacteria [311]. Probiotic‐fermented herbal medicine, as an important method for maintaining gut microecology, holds great potential for application. But its underlying mechanisms still require further research.

### 5.5 Emerging Therapies

When reviewing the relationship between gut microbiota dysbiosis and diseases, a large group of metabolic disorders have been found that their potential pathogenic mechanisms related to microbial metabolites. For example, tryptophan metabolites can regulate T cell stemness and enhance immune effects in cancers [312]. Microbial metabolites derived from methylamine, branched‐chain amino acid metabolism, and carbohydrate fermentation may play a significant role in the pathogenesis of NAFLD [313]. And abnormal BAs metabolism exacerbates the progression of NAFLD [314, 315]. SCFAs are the most studied gut microbiome metabolites. They stimulate the production of 5‐HT, regulate the maturation of microglial cells in the CNS [316], and are associated with CNS diseases such as PD^152^ and AD [317]. Dietary supplementation with SCFAs can promote metabolic coupling between astrocytes and neurons, alleviating the pathological progression of AD [317]. Butyrate, as an immunomodulator in the gut and other systems, is closely related to IBD [318, 319]. Although these metabolites have various therapeutic effects, their poor pharmacokinetic properties have remained a bottleneck limiting their translational applications. To overcome the characteristic of low molecular weight SCFAs being easily cleared, Babita et al. made significant progress in applying SCFAs for melanoma treatment through nanoparticle delivery [320]. Using genetic engineering strategies to enhance the bioavailability of microbial metabolites in the body is a major approach to address their pharmacokinetic challenges.

In the field of microbiome therapy, applying drug development principles (including dose–response, pharmacokinetics, and clearance) is particularly challenging. Because they consist of living organisms and confined to the gut. They may replicate or lose bioactivity during passage through the GI tract. Detecting the infused therapeutic biologics in the context of the host microbiome is also challenging. As sampling from the intact host's gut is not possible except for collecting feces, making it especially difficult to assess the vitality and activity of the microorganisms. With the development of synthetic biology, new strategies for treating various diseases. Engineered microorganisms have been developed in metabolic disorders and cancer. For example, engineering microorganisms to produce cytokines, inhibit pathogenic bacteria, and deliver therapeutic agents for targeted delivery has yielded positive preclinical trial results [321, 322, 323]. The engineered Escherichia coli strain has been used in the treatment of NAFLD. This strain converts systemic ammonia into l‐arginine in a mouse model. In a human study, 52 healthy subjects showed good tolerance and demonstrated dose‐dependency [324]. The modified *Escherichia coli* strain, PAS638, can persist long term in the mammalian intestine and exert its effects, serving as an in vivo diagnostic agent for inflammation [325]. In a mouse model, the inoculation of a bacterial consortium containing the urease gene also achieved treatment of hyperammonemia [326]. Engineered probiotics are also applied in cancer treatment [327]. These findings enable the implementation of more precise treatments using engineered microorganisms. However, many practical challenges remain to be overcome, including preventing gene transfer between microorganisms and off‐target effects.

### 5.6 Precision Medicine Approaches

Due to the unique characteristics of an individual's microbiome, there are differences in the degree and direction of their responses to diet and disturbances. The human microbiome is closely related to overall health, leading to the ongoing development of microbiome‐based therapies in disease treatment, such as FMT for *Clostridium difficile* infection. However, these microbiomes are highly complex, dynamic, and personalized ecosystems, exhibiting significant interindividual variability in terms of species composition [328] and abundance profiles [329]. This interindividual variability may lead to unexpected consequences of universal microbiome interventions, rendering them ineffective or even harmful. To be effective, this treatment approach may require personalized insights into an individual's genetics, diet, gut microbiome, and other environmental factors that could be involved. Therefore, precision treatment strategies based on microbiome‐driven personalized therapy have been developed. This approach using the characteristics of an individual's gut microbiome or the gut microbiome associated with a specific disease to tailor treatment plans. By utilizing new computational methods that characterize the dynamics of human‐associated microbiomes [330], along with metagenomic measurements and other techniques, individual differences can be identified to develop personalized diagnostic and therapeutic plans. It is aim to maximize the safety and effectiveness of treatment outcomes. In a study involving approximately 300 children with ASD, researchers developed customized synbiotics for each individual based on metagenomic analysis of fecal samples and personal health and dietary surveys. The overall effect of precise synbiotic supplementation on autism shows a positive response, including improvement in GI symptoms [58]. A new therapeutic approach to preventing and controlling diseases is to regulate host–microbiome interactions through personalized nutrition plans.

Besides, using multiscale modeling to explore the mechanistic chain and causal relationships between the microbiome and diseases, along with techniques like machine learning (ML), to create personalized models [331] is also a significant shift from traditional diagnostics to personalized interventions. With the rapid development of AI, applying this technology to explore the complex datasets of the gut microbiome and extract valuable information is of significant importance for personalized medicine and disease treatment [332]. AI includes various technologies such as ML, deep learning (DL), and natural language processing [333]. AI technology has been used in the fields of medical imaging [334], genomic analysis [335], and drug development [336]. Currently, AI technologies such as ML can effectively infer changes in the gut microbiome composition associated with diseases and identify phenotypic variations based on more personalized features [337]. In addition, AI technology can be used to simulate the infant microbiome, predict the dynamic changes of species in the gut, and track how they evolve with the infant's development, providing early indications for neonatal developmental disorders [338]. ML and DL, due to their powerful predictive and information‐processing capabilities, have been used to study the relationship between microbiome composition and its connection to phenotypes [339].

However, it is worth noting that the human gut microbiota is complex. The community composition in microbial data exhibits heterogeneity and the application of AI technology lacks standardized specialized processes [340]. As a result, applying AI to the analysis of microbial data still presents certain challenges [341]. Moreover, in clinical applications, using AI technology to interpret the intestinal flora requires attention to the interpretability of the analysis model [342]. Furthermore, ethical and moral factors must be fully considered to ensure that the use of AI in microbiome research is safe and fair [343]. In the future, with the advancement of AI technology, it may be able to quickly process and interpret large amounts of gut microbiome and metabolic data. It will help researchers explore specific microbial characteristics/patterns and potential biological mechanisms. Finally, personalized treatment of diseases may come true based on above research.

## 6 Challenges and Future Directions

The significant interindividual variability in the human microbiome presents substantial challenges for studying the specific profiles of gut microbiota. With the development of omics, through a deeper integration of metagenomics, metaproteomics and metabolomics to characterize the characteristics of individual microbiomes comprehensively. It is possible to reveal the mechanisms behind individual differences. Moreover, large‐scale cohort studies and the application of AI technology is expected to establish a personalized microbiome prediction model to provide new perspectives for precision medicine. Standardized methods for microbiome research are the foundation for establishing precise diagnosis and treatment. Currently, the methods for microbiome research are not fully unified, which limits the comparability of results across different studies. Developing a standardized experimental design, sample collection, data processing, and analysis is essential for ensuring the reliability and comparability of research outcomes. Additionally, the development of novel high‐throughput and high‐sensitivity technologies, such as microbial single‐cell sequencing and spatial transcriptomics, will enable deeper insights into the structure and function of microbiomes. Currently, there are two main methods for gut microbiome sequencing: 16S rRNA‐based sequencing and whole‐metagenome shotgun sequencing (WGS). However, due to the short read lengths of 16S rRNA and WGS sequencing, there is fragmentation of the microbial genomes [344]. To address this, more advanced third‐generation sequencing technology is used to detect bacterial metagenomes. Compared with previous methods, it can detect longer fragments. In addition, Single Molecule Real‐Time Sequencing of Pacbio used for detecting the gut microbiome metagenome has the ability to efficiently assemble bacterial genomes [345]. Next‐generation sequencing technology is characterized by its ability to generate large amounts of data in a short period of time [346]. To further determine the spatial distribution of metabolites, the application of spatial metabolomics enhances the understanding of the spatiotemporal characteristics of biological processes. Furthermore, the application of organs and organoids chip has important implications for the understanding of the gut–organ axis.

Exploring the causal relationships and correlation between the microbiome and diseases is the core focus of microbiome analysis. Current evidence suggests that the human gut microbiome is closely associated with various diseases, but the causal relationships and pathogenic mechanisms require further investigation. In the future, through animal models, large‐scale population cohort studies combined with evidence‐based medicine, multiomics data integration, and AI technology will enable to more accurately reveal the role of the microbiome in the occurrence and development of diseases. Combining phenomics and environmental factor analysis will help elucidate the complex network of host–microbiome interactions. This will provide new targets for disease prevention, diagnosis, and even personalized treatment. With a deeper understanding of the relationship between the microbiome and diseases, novel microbiome‐targeted drugs are expected to be developed. These may include probiotics, prebiotics, bacteriophages, microbial metabolites to achieve therapeutic effects by targeting specific microbial communities, functional genes, or metabolic pathways. Building on FMT, standardized microbiota transplantation techniques will provide innovative treatments for refractory diseases.

**FIGURE 5:**
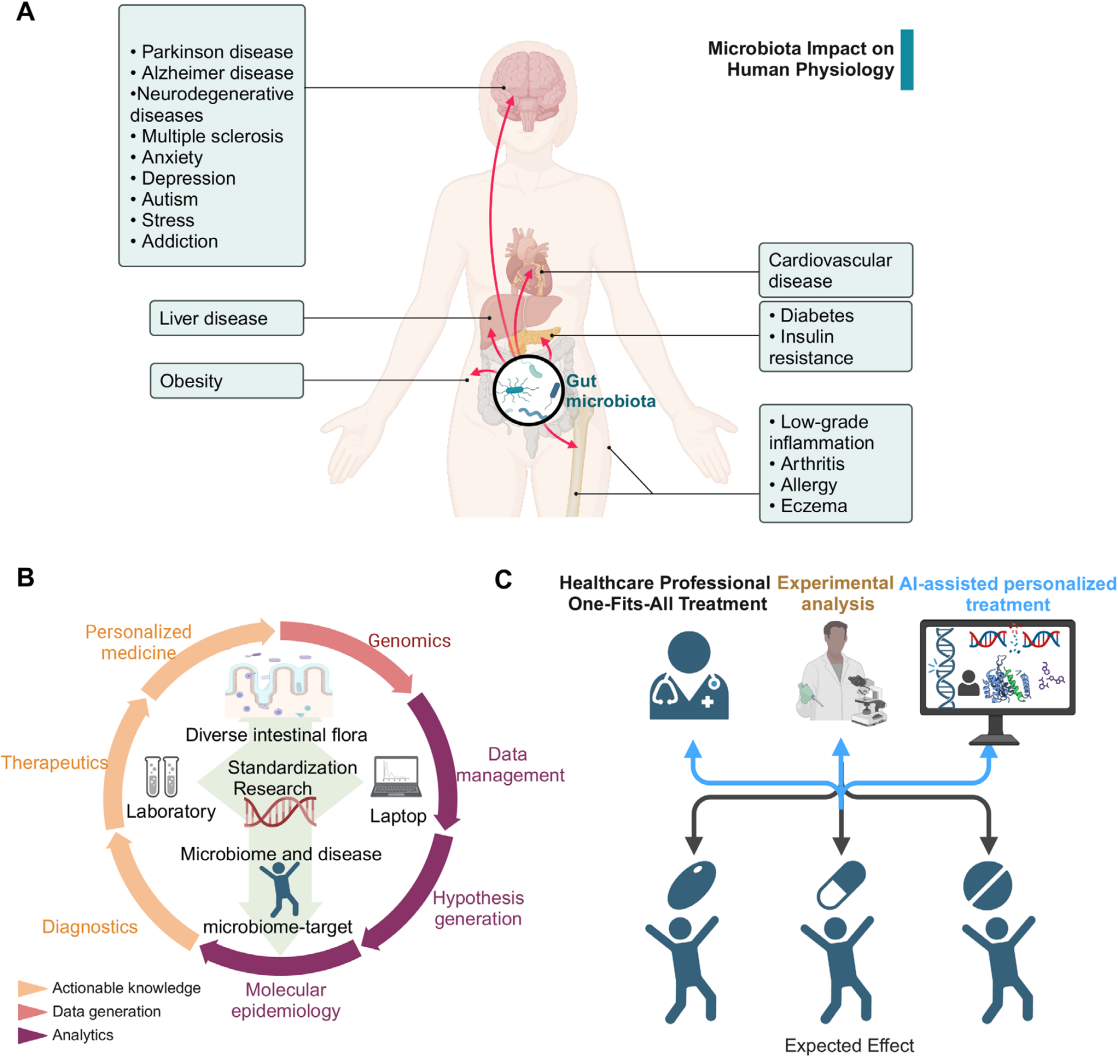
Establishing personalized precision diagnosis and treatment models based on the close relationship between gut microbiota dysbiosis and disease. (A) The onset of various systemic diseases is closely associated with the imbalance of gut microbiota homeostasis. (B) The challenges of establishing personalized precision treatment systems based on gut microbiota. The process includes obtaining information on gut microbiota dysbiosis, developing standardized analytical workflows, and achieving targeted interventions based on individual variations. All steps require a comprehensive approach, spanning from the interpretation of microbiome data to epidemiological analyses, as well as diagnostic and therapeutic applications to the standardized synthesis of precision drugs. A closed‐loop system supported by big data must be developed to enable precision diagnosis and treatment. (C) Precision diagnosis and treatment supported by big data can be broadly divided into three components: one‐on‐one clinical diagnosis and treatment for patients, laboratory sample collection and analysis, and AI‐assisted personalized treatment (created in BioRender. Yang, X. (2025) https://BioRender.com/undefined).

In conclusion, the future of microbiome research is full of opportunities and challenges. Through interdisciplinary collaboration, continuous innovation in research methodologies, and relentless exploration, microbiome studies will make greater contributions to advancing human health (Figure 5).

## Author Contributions

*Review design*: Yao Shen, Nairui Fan, and Guang Wang. *Data collection and figure preparation*: Yao Shen, Nairui Fan, Shu‐xia Ma, and Xin Cheng. *Manuscript revision*: Xuesong Yang and Guang Wang. All authors have read and approved the final manuscript.

## Ethics Statement

The authors have nothing to report.

## Conflicts of Interest

The authors declare no conflicts of interest.

## Data Availability

The authors have nothing to report.
